# Quantum bioinformatics: a systematic review of methods, trends, and challenges

**DOI:** 10.1093/bib/bbag383

**Published:** 2026-07-17

**Authors:** Mehdi Khalaj, Steven Rayan, Lingling Jin

**Affiliations:** Department of Computer Science, University of Saskatchewan, 110 Science Pl., Saskatoon, SK, S7N 5C9, Canada; Centre for Quantum Topology and Its Applications (quanTA), University of Saskatchewan, 110 Science Pl., Saskatoon, SK, S7N 5C9, Canada; Department of Mathematics and Statistics, University of Saskatchewan, 106 Wiggins Road, Saskatoon, SK, S7N 5E6, Canada; Department of Computer Science, University of Saskatchewan, 110 Science Pl., Saskatoon, SK, S7N 5C9, Canada

**Keywords:** quantum computing, bioinformatics, quantum bioinformatics, systematic review

## Abstract

Modern bioinformatics faces escalating challenges stemming from both the inherent computational hardness of many fundamental problems and the rapidly growing scale and complexity of biological data, increasingly limiting the effectiveness of classical computational approaches. Quantum computing has emerged as a promising paradigm for addressing these challenges by enabling alternative problem representations and novel search strategies for exploring complex solution spaces. This systematic review provides a structured overview of the emerging field of quantum bioinformatics and aims to supplement recent reviews on this topic in the journal by providing an updated and structured synthesis of current research. We systematically collect and organize existing studies across 10 bioinformatics domains to identify research trends, dominant themes, and recurring methodological patterns. The review examines quantum and hybrid quantum-classical approaches, problem formulations, and encoding strategies, with particular attention to the constraints of noisy intermediate-scale quantum devices, including noise, limited scalability, and the need for error mitigation. We further synthesize reported limitations, open challenges, and prospective research directions.

## Introduction

### Background

Modern biological science increasingly relies on bioinformatics to tackle complex problems, such as protein folding and drug discovery, driven by the complexity of biological systems and the rapidly growing volumes of data that exceed the capabilities of traditional wet-lab techniques alone. These challenges demand sophisticated computational biology approaches to generate candidate hypotheses and mechanistic insights, which can then be experimentally validated through wet-lab studies to establish biological relevance.

Across bioinformatics domains, classical computational methods face limitations in scalability and accuracy due to the exponential growth of biological data and the inherent hardness of some problems. Tasks such as *de novo* genome assembly and multiple sequence alignment are Nondeterministic Polynomial (NP)-hard, and established assembly methods struggle with repeats, errors, or haplotype complexity, while alignment workloads can exceed classical high-performance computing capacity [1–3]. In drug discovery, classical docking and simulation pipelines are challenged by the vast chemical search space and difficulties in modeling molecular interactions accurately [4]. Protein structure prediction is NP-complete, and neither classical molecular docking nor deep networks can feasibly explore the vast and complex conformational landscape [5]. Gene regulatory network inference and disease-gene prioritization involve exponential state spaces, limiting classical network models [6, 7]. Motif identification and sequence similarity search are often NP-hard or memory-intensive, with runtimes growing rapidly with database size [8–10]. Gene-expression analysis and feature selection must manage high dimensionality and noise, which can cause classical optimization to converge early. Multi-omics integration compounds these issues by creating large, cross-modal feature spaces with complex dependencies [11]. Protein design is NP-hard due to the exponential sequence-structure space, and phylogenetic reconstruction becomes computationally intractable as tree topologies grow super-exponentially with taxa. Machine learning approaches in these domains are often constrained by high dimensionality, nonlinear feature interactions, and limited or biased training datasets, increasing overfitting risk and poor generalization. Collectively, classical computing faces increasing difficulty efficiently handling the high dimensionality and combinatorial complexity of modern bioinformatics problems.

Quantum computing (QC) offers new computational approaches and has shown early promise in addressing complex biological problems [12]. By leveraging principles of quantum mechanics, such as superposition, entanglement, and quantum tunneling, QC can outperform classical computers for certain problem classes. These effects let algorithms reformulate some bioinformatics problems into alternative representations, enabling more efficient exploration of large search spaces and improved modeling of complex, high-dimensional biological relationships.

Collectively, quantum approaches may provide complementary strategies for some limitations of classical bioinformatics methods. However, the current literature remains fragmented, spanning diverse applications, methodologies, and levels of technical maturity. This review systematically gathers, organizes, and synthesizes existing studies into an integrated view of quantum bioinformatics, structuring the literature into meaningful categories to identify dominant themes and methodological patterns at the intersection of quantum computing and bioinformatics.

The scope does not include independent benchmarking or performance validation; any claims of quantum utility or advantage are presented solely as stated by the original authors, without additional interpretation or verification.

### Motivation

This systematic review provides a structured overview of the scope and distribution of research within the field of Quantum Bioinformatics. We first examined existing reviews of this rapidly evolving field. Although the systematic review mapping by [13] provides a valuable high-level catalogue of quantum-bioinformatics methods, it lacks quantitative analytics, domain-specific qualitative insights, or a thematic synthesis. The taxonomy-oriented article [14] systematically distinguishes quantum biology from quantum bioinformatics but does not examine methodological trends or assess hardware readiness. While prior studies have demonstrated proof-of-concept quantum advantages for selected bioinformatics applications [15] and surveyed major computational bottlenecks in the field [12], a comprehensive and systematic review of the literature at the intersection of quantum computing and bioinformatics remains lacking. Other reviews adopt a narrower focus on specific quantum techniques or domains; for example, quantum natural language processing (QNLP) applied to genomic sequence analysis, protein structure prediction, and drug discovery [16] or quantum-mechanical formulations of drug discovery problems [17].

Our review offers an integrated examination of quantum bioinformatics, combining quantitative descriptive analytics, qualitative synthesis across 10 bioinformatics domains, thematic analysis of noisy intermediate-scale quantum (NISQ) era challenges (noise, scalability), and quantum encoding strategies, and a structured assessment of current limitations and future research directions.

### Objectives

This review aims to synthesize current knowledge on the specified research topics: to characterize research trends, dominant themes, and the current state of quantum bioinformatics, and to identify and categorize quantum computing methods and algorithms applied to bioinformatics problems. The review further examines the bioinformatics tasks explored using quantum and hybrid quantum-classical approaches, and analyzes how studies address hardware constraints, particularly noise and error mitigation on NISQ devices. In addition, it investigates strategies for encoding biological information into quantum-readable representations, and synthesizes the limitations, open challenges, and future research opportunities in the field.

## Methodology

This systematic review was conducted and reported in accordance with the Preferred Reporting Items for Systematic Reviews and Meta-Analyses (PRISMA) [18] guidelines. This section outlines the planning and execution of the review, including the criteria and procedures used to identify, select, and map the analyzed literature.

### Information sources

The sources of articles should be reliable, as they will affect how well the review is conducted. Given the interdisciplinary nature of this research, we searched three information sources to capture the broad range of relevant literature. Scopus was used as the initial source for its extensive multidisciplinary coverage. The finalized search was then adapted in Web of Science, another important multidisciplinary database, to identify additional studies that may not be indexed in Scopus. We also conducted a complementary Google Scholar search to capture studies not indexed in traditional databases. The search was completed on 31 October 2025.

### Inclusion and exclusion criteria

To ensure that only relevant and high-quality studies were included, all retrieved articles were screened according to predefined inclusion and exclusion criteria. Eligible studies had to present an original theoretical or practical application of a quantum or hybrid algorithm to a bioinformatics problem, ranging from mathematical analyses to proof-of-concept implementations, while reviews and papers in which bioinformatics was not the central focus were excluded. The primary research studies included were published between 2021 and 2025. The full set of inclusion and exclusion criteria is presented in Table 1.

**Table 1:** Inclusion and exclusion criteria.

**Inclusion**	**Exclusion**
Research studies published between 2021 and 2025	Research studies published before 2021
Peer-reviewed journals and conference papers	Magazines, review papers, and books
Articles are written in English	Articles are not written in English
Articles that contain at least four pages	Articles that have fewer than four pages
Subject areas: Bioinformatics, Computer Science, Computational Biology, Immunology and Microbiology, and Pharmacology	Other areas: Biochemistry, Chemistry, Biophysics, Physical Chemistry, Chemical Simulation, Quantum Chemistry, and Molecular Properties

### Search strategy

This review follows PRISMA guidelines for transparent reporting and systematic screening; the search strategy targeted representative rather than fully exhaustive coverage, given the breadth and rapid growth of this interdisciplinary field, where terminology and venues are highly diverse. Rather than collecting every publication, we identified a representative set of articles capturing key research activity at the quantum-bioinformatics intersection. We employed multi-database searches with inclusive query terms, applied predefined eligibility criteria, and supplemented retrieval through reference screening. The corpus was validated against key studies and recent reviews to confirm coverage across major domains, methodologies, and maturity levels, minimizing selection bias. Title-based queries improved precision in a terminology-inconsistent field and ensured retrieved studies explicitly focus on both quantum computing and bioinformatics, complemented by multiple databases and reference screening to reduce potential omissions, although some relevant studies may have been missed if key terms appeared only in abstracts or keywords.

An example search string is in the supplemental document. The search included QC-related keywords such as “quantum,” “quantum computing,” “quantum algorithm,” “quantum annealing,” “quantum machine learning,” “variational quantum,” “quantum circuit,” and “quantum simulation.” These were paired with bioinformatics-related keywords including “bioinformatics,” “genomics,” “proteomics,” “transcriptomics,” “sequence alignment,” “genome assembly,” “protein folding,” “protein structure,” “molecular docking,” “drug discovery,” “gene expression,” “protein interaction,” and “computational biology.”

### Review process

**Figure 1:**
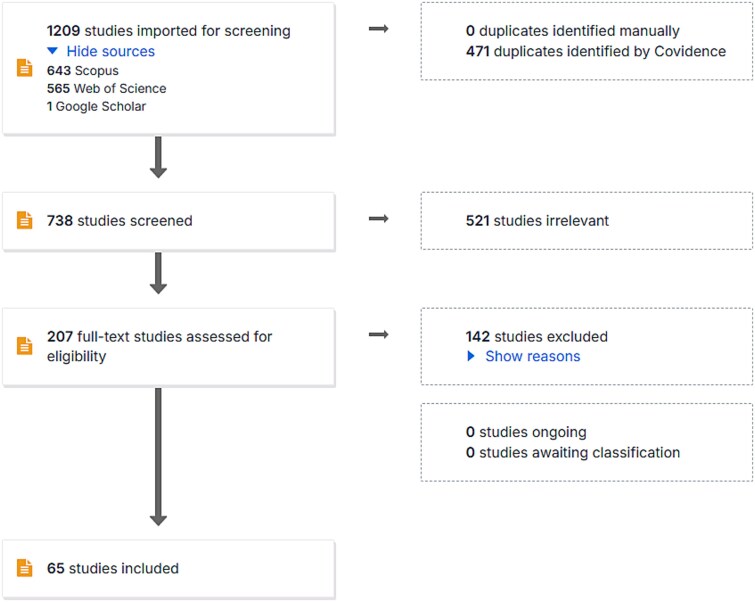
PRISMA flowchart.

We used Covidence [19], an online systematic review management platform, to support the study selection process. Citations retrieved from the three databases were imported into Covidence, where automatic de-duplication was performed. Two reviewers (MK and LJ) independently screened the titles and abstracts, followed by full-text screening, against inclusion and exclusion criteria. These eligibility criteria were clearly specified and embedded within the Covidence platform to minimize disputes. Articles deemed relevant or marked as “unsure” by either reviewer were carried forward to the next screening stage. Discrepancies at both the title/abstract and full-text stages were identified by the software and resolved through discussion and consensus. Reasons for exclusion at the full-text review stage were documented. The overall study selection process is summarized in a PRISMA flow diagram (Fig. 1).

### Data extraction

A data extraction form was developed and piloted in Covidence to ensure consistency. One reviewer (MK) performed the extraction, and a second reviewer (LJ) verified all entries for accuracy and completeness. For each study, we extracted bibliographic (e.g. year, country, and venue), methodological (e.g. QC methods, encoding strategies, and technology readiness), and thematic information (e.g. domain, objectives, contributions, data types, key findings, limitations, and future directions). The extracted data were then synthesized to provide a comprehensive overview of QC applications across bioinformatics domains.

## Data analysis and results

### Descriptive analysis

**Figure 2:**
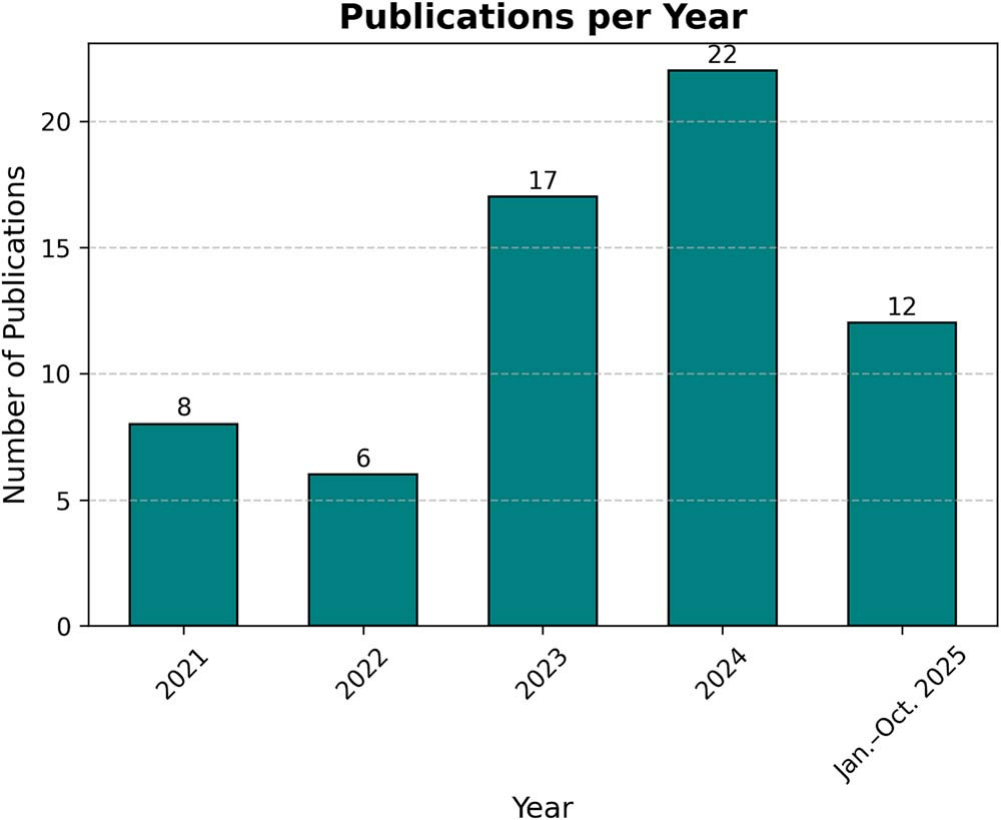
Publication trends in quantum bioinformatics from January 2021 to October 2025.

**Publication trends over time:** the publication trend (Fig. 2) shows a steady increase in quantum bioinformatics research over the past 5 years. Research activity was sparse during 2021–2022, followed by a notable rise in 2023 and a peak in 2024. The lower publication count observed for 2025 is likely attributable, at least in part, to incomplete data coverage, as records extend only through October. Consequently, no definitive conclusions can be drawn regarding how the full-year output for 2025 will compare with that of 2024. Overall, the temporal pattern suggests a clear upward trajectory, reflecting growing research activity and sustaining interest in the field.

**Figure 3:**
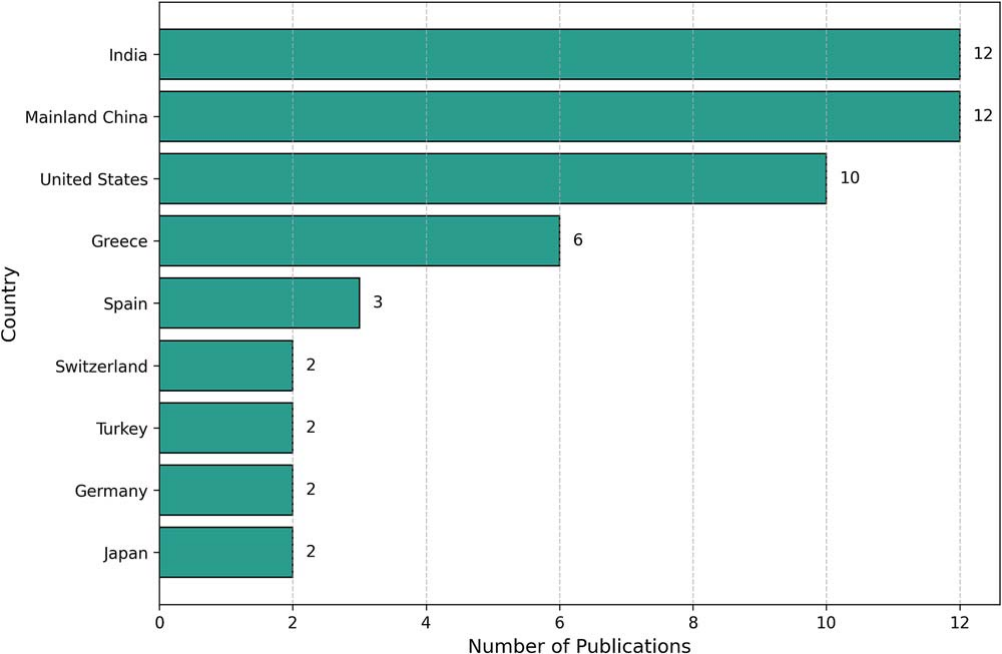
Publications by country (counts >1).

**Leading countries (by country of the corresponding author):** the geographic distribution of publications (Fig. 3) shows that quantum bioinformatics research is dominated by contributions from India, Mainland China, and the United States, which together account for most of the output. Several European countries, including Greece, Spain, Germany, and Switzerland, also contribute consistently, with additional participation from other regions, indicating that the field has started to grow across many countries worldwide. The countries with just a single publication are not shown in the diagram.

**Figure 4:**
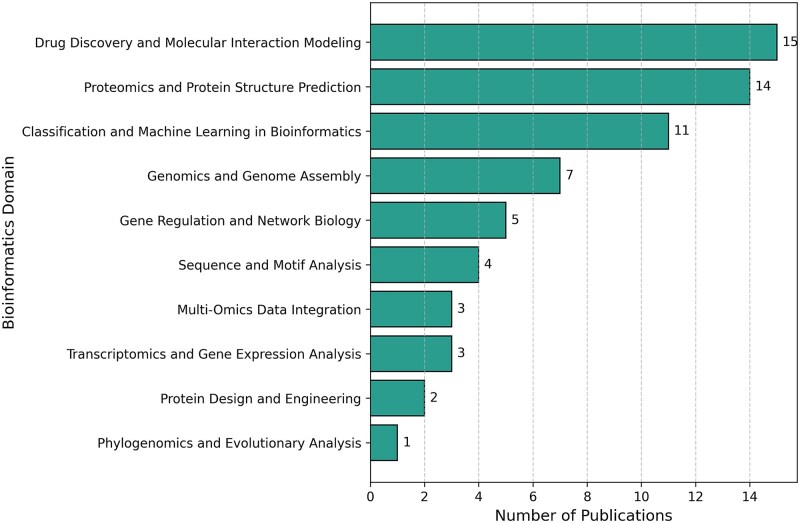
Publication by bioinformatics domains.

**Bioinformatics domains:** the 10 bioinformatics domains were defined based on commonly recognized task types and problem formulations in the field, enabling consistent grouping of studies by their primary biological objective. In cases where studies span multiple areas, each was assigned to the domain that best reflects its main contribution to maintain clarity and comparability. The distribution of application domains (Fig. 4) shows that current quantum bioinformatics research is concentrated in a few areas. Drug discovery and protein structure prediction emerge as the most popular and extensively studied domains, reflecting both their central importance in computational biology and their perceived suitability for quantum optimization and quantum simulation paradigms. By contrast, multi-omics integration, gene expression analysis, protein design, and phylogenetics remain less represented, suggesting opportunities for future work in quantum methods and hardware development.

**Figure 5:**
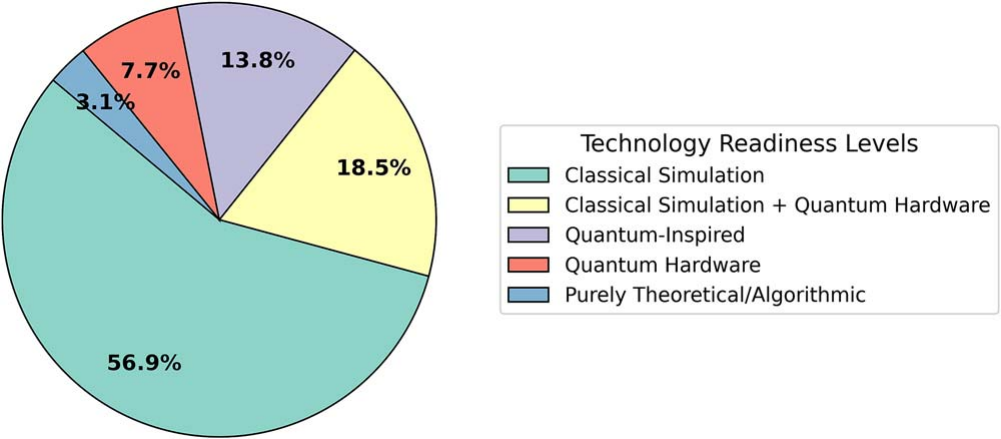
Distribution of publications by technology readiness level.

**Technology readiness level (TRL):** the TRL distribution (Fig. 5) shows that a majority (56.9%) relies on **quantum simulation**, indicating that current work focuses primarily on verifying algorithmic feasibility rather than achieving hardware execution. A smaller group (3.1%) presents **purely theoretical frameworks** without implementation, while **quantum-inspired methods** (13.8%) leverage principles of quantum computation to improve classical models. Only 7.7% of studies demonstrate execution on **quantum hardware**, reflecting limited but growing engagement with real devices. These studies run algorithms on current-generation hardware (10–100+ qubits) to evaluate noise resilience, scalability, and performance trade-offs. The remaining 18.5% utilize **hybrid quantum simulation + hardware** workflows, highlighting a transitional phase in which candidate algorithms are validated on simulators before limited deployment on NISQ processors. Overall, the landscape reflects an exploratory field gradually progressing toward practical hardware experimentation.

**Real hardware usage:** as shown in Table 2, out of 65 reviewed studies, 17 (9.2%) utilized real quantum hardware, and their analysis demonstrates a clear and steady evolution in QC maturity within the field of bioinformatics. Early studies (2021–2022) predominantly relied on first- and second-generation quantum hardware, including D-Wave’s 2000Q annealer and IBM’s small-scale gate-based systems. Owing to their limited qubit counts and restricted coherence, these platforms were used primarily for proof-of-concept and feasibility demonstrations. From 2023 onward, studies increasingly incorporated more advanced platforms, including IBM Falcon/Eagle systems, D-Wave Advantage, and devices from IonQ, Rigetti, and Quantinuum, enabling higher qubit counts, improved connectivity, and more stable execution. By 2024–2025, several studies benchmarked algorithms on 100+ qubit processors, reflecting a shift from purely conceptual testing to more substantive hardware validation and hybrid quantum-classical workflows. Overall, the trend indicates gradual but steady progress toward practical hardware deployment in quantum bioinformatics research.

**Table 2:** Summary of real quantum hardware usage across the reviewed studies.

**Study**	**Year**	**Provider**	**Hardware**	**Number of qubits**	**Processor**
[4]	2021	IBM	Superconducting transmon	5	Canary
[20]	2021	IBM	Superconducting transmon	27	Falcon
[21]	2021	D-Wave	Annealer	2000+	2000Q
[22]	2021	D-Wave	Annealer	2000+	2000Q
[23]	2022	IBM	Superconducting transmon	27	Falcon
[24]	2022	D-Wave	Annealer	5000+	Advantage
[25]	2022	D-Wave	Annealer	2000+	2000Q
[26]	2023	Rigetti	Superconducting transmon	32	Aspen10
		IBM	Superconducting transmon	5	Canary
[27]	2023	IBM	Superconducting transmon	27	Falcon r4
		IonQ	Trapped-ion	11	trapped-ion
[28]	2023	IBM	Superconducting transmon	27	Falcon
		IBM	Superconducting transmon	16	Falcon
[29]	2023	IBM	Superconducting transmon	27	Falcon
		IBM	Superconducting transmon	27	Falcon
[30]	2023	Quantinuum	Trapped-ion	20	H1-1 trapped ions
		IBM	Superconducting transmon	16	Falcon
[31]	2024	IBM	Superconducting transmon	127	Heron
		IBM	Superconducting transmon	133	Eagle
[32]	2024	D-Wave	Annealer	5000+	Advantage
[33]	2024	D-Wave	Annealer	5000+	Advantage
[34]	2024	D-Wave	Annealer	5000+	Advantage
		IBM	Superconducting transmon	127	Eagle
[35]	2025	IBM	Superconducting transmon	127	Brisbane

**Figure 6:**
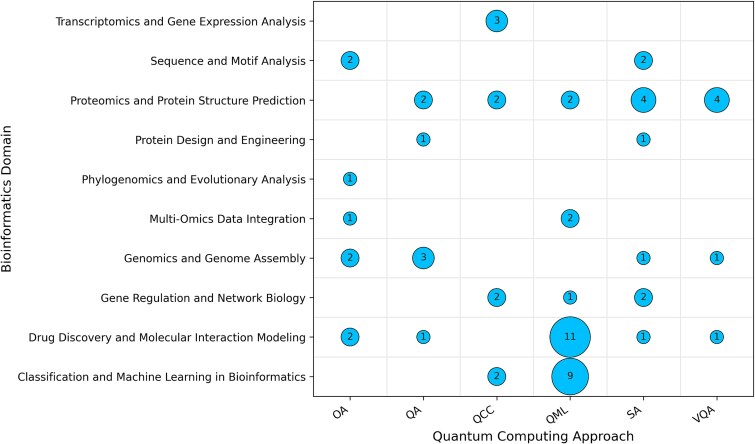
Co-occurrence: quantum computing approaches × bioinformatics domains.

**Co-occurrence between quantum approaches and bioinformatics domains:** a co-occurrence analysis (Fig. 6) reveals the relationship between different QC paradigms and their application domains in bioinformatics. Quantum Machine Learning (QML) methods dominate the landscape. This widespread use indicates that QML methods are viewed as the most versatile framework for handling complex, data-rich problems such as pattern recognition, disease classification, and molecule-target prediction.

Variational Quantum Algorithms [36], particularly the Variational Quantum Eigensolver (VQE) [37], are used mainly in protein folding simulations and protein structure prediction, where hybrid quantum-classical optimization is well aligned with structure-optimization tasks.

Quantum Optimization Algorithms (OA), including the Quantum Approximate Optimization Algorithm (QAOA) [38] and Quadratic Unconstrained Binary Optimization (QUBO) formulations [39], are primarily applied to bioinformatics problems with an underlying combinatorial structure. These applications include genome assembly, sequence/motif analysis, phylogenetic tree construction, multi-omics integration, and molecular docking. Quantum Annealing (QA) [40] has been applied in similar domains, particularly for graph-based problems and clustering tasks.

Studies categorized under Fundamental Quantum Computing Concepts (QCC) focus on theoretical modeling of biological processes and are most common in transcriptomics and gene expression analysis, gene regulation and network biology, and protein structure prediction.

Finally, gate-based quantum search algorithms (SA), including Grover’s search [41] and quantum-walk search [42], are used in limited yet targeted bioinformatics areas, particularly in proteomics and sequence analysis. These include applications in protein structure prediction and gene regulatory network inference, where quantum search provides theoretical speedups for motif discovery, pattern matching, and data retrieval.

### Qualitative analysis

In this subsection, we provide a qualitative analysis of the application of quantum algorithms to diverse bioinformatics problems. The reviewed studies are categorized into 10 bioinformatics domains, and the specific quantum techniques used in each domain are examined. A detailed, study-by-study description of all the works included is provided in the Supplementary Material.

#### Genomics and genome assembly

Classical genome assemblers based on Overlap-Layout-Consensus (OLC) and de Bruijn graph (DBG) models face fundamental limitations with long repetitive elements, high error rates, and complex haplotypes [2]. While hybrid short- and long-read methods improve accuracy, they remain computationally intensive and constrained by the architectural limits of classical computing [3]. In parallel, the rapid growth of genomic data has pushed sequence-alignment demands beyond the capacity of classical infrastructures [1].

To address these challenges, recent studies reformulate genome assembly and alignment as quantum optimization problems, primarily using QUBO models and hybrid workflows. Sarkar et al. [21] present an end-to-end quantum formulation of *de novo* genome assembly by mapping the OLC problem to QUBO, showing that quantum annealing achieves optimal read ordering while QAOA provides approximate but consistent solutions. Boev et al. [22] and Nałęcz-Charkiewicz et al. [24] similarly apply quantum annealing to QUBO-based assembly formulations, demonstrating feasibility on small synthetic and limited real datasets, though scalability is constrained by hardware noise and connectivity. Fang et al. [43] introduce a divide-and-conquer hybrid approach that combines classical preprocessing with VQE to solve smaller QUBO subproblems, enabling execution on limited qubit systems. Varsamis et al. [44] propose a hybrid framework that integrates QAOA-based Max-Cut, quantum walks, and Hamiltonian path solvers for genome assembly, but results remain simulation-based. Chen et al. [32] apply a hybrid quantum-classical pipeline to haplotype-resolved assembly, using classical solvers for large instances and quantum annealing for refinement. Finally, Varsamis et al. [45] propose a gate-based method for reference-guided DNA alignment, validated in simulation on small sequences.

#### Classification and machine learning in bioinformatics

Classical machine learning and deep learning are central to bioinformatics tasks such as disease subtype classification, biomarker discovery, and mutation prediction. However, biological data are typically high-dimensional and heterogeneous, with features far exceeding samples. This imbalance drives overfitting and high optimization cost, and conventional models struggle to capture the entangled correlations among genes or proteins that reflect complex biological processes.

Researchers apply quantum machine-learning methods to biomedical classification, prediction, and biomarker discovery, encoding high-dimensional features into quantum states to explore complex feature relationships more efficiently and improve accuracy. Quantum-inspired and hybrid quantum-classical deep-learning pipelines have been increasingly applied to disease classification and risk prediction, including white blood cell classification [46], cardiovascular disease prediction [47], diabetes prediction [48], sarcopenia risk assessment [49], and heart disease prediction [50]. These approaches typically integrate classical feature extraction with QML models such as Quantum Support Vector Machine (QSVM), Quantum Neural Network (QNN), Quantum K-Nearest Neighbor classifier (QKNN), or quantum-enhanced attention mechanisms. Cancer-related applications are prominent, with quantum or hybrid approaches proposed for cancer prediction using gene-expression data [51], brain tumor classification [52], biomarker discovery from multi-omics datasets [53], and renal cancer biomarker classification [54], where quantum feature maps and variational classifiers show competitive or improved performance on reduced datasets. Protein and sequence analysis is also explored, including Quantum Tensor Networks [55] for protein classification [56] and a Quantum LSTM model for SARS-CoV-2 mutation prediction [57], both demonstrating parameter efficiency or performance gains in simulation.

#### Drug discovery and molecular interaction modeling

Classical computer-aided drug discovery methods, including molecular docking, binding-affinity prediction, and compound–protein interaction modeling, face fundamental scalability limits, as the deep learning and molecular dynamics pipelines they rely on are data- and resource-intensive and often require costly screening of millions of compounds. These constraints slow early-stage discovery and limit exploration of the vast chemical space [4].

Researchers explore quantum and hybrid quantum-classical strategies for key drug-discovery bottlenecks, mostly in simulation-based settings. Hybrid architectures are common, including Quantum Generative Adversarial Network (QGAN) for small-molecule generation [4], quantum autoencoders for molecular representation learning [58], and parameterized quantum circuits (PQCs) enhanced deep-learning models for binding-affinity prediction [59–61], which often achieve comparable or improved performance with fewer parameters than classical counterparts. Quantum kernel and variational methods are widely applied to virtual screening, bioactivity prediction, and compound–protein interaction tasks using Quantum Support Vector Classifiers (QSVCs) [62], Variational Quantum Classifier, and QSVM models [26, 28, 63–65], typically after aggressive feature reduction to meet qubit limits. Several works reformulate docking and interaction problems as quantum search or optimization tasks, employing Grover’s algorithm and SWAP-test [66] similarity measures [35], QUBO-based quantum annealing [34], or QAOA and its variants [67], with annealing often outperforming gate-based methods under NISQ constraints. Other studies integrate VQE-derived quantum features with classical graph neural networks for molecular property prediction [68] or propose enhanced QNN architectures with reinforcement learning and noise mitigation for drug-discovery applications [69].

#### Proteomics and protein structure prediction

This domain involves predicting a protein’s 3D conformation from its amino acid sequence over an astronomically large search space, a problem that is classically NP-complete and infeasible to explore exhaustively even on the most powerful supercomputers [5]. Traditional approaches such as molecular dynamics, genetic algorithms, and deep learning struggle to balance accuracy and efficiency, often relying on heuristics that fail for large or complex proteins.

By formulating folding as a quantum optimization, these approaches embed quantum effects directly into the modeling process for more scalable predictions within NISQ constraints. Several works reformulate folding as a search or optimization problem, applying Grover’s search to hydrophobic-hydrophilic (HP) lattice models to identify low-energy conformations with expected quadratic speedups in simulation [70–72]. Hybrid and variational methods are also common, including Conditional Value-at-Risk Variational Quantum Eigensolver (CVaR-VQE) [73] based folding algorithms [20, 31], quantum annealing formulations of Ising or QUBO models [25], and Digitized Counterdiabatic protocols that improve convergence and stability on hardware devices for small peptides [30]. Quantum walks are explored as a modeling tool for protein folding dynamics and conformational transitions, both in quantum-inspired theoretical frameworks [74] and hybrid quantum-classical algorithms [23, 75] combined with Metropolis sampling [76]. Machine-learning-based approaches include Quantum Convolutional Neural Networks (QCNNs) for protein distance prediction [77] and quantum reinforcement learning [78] for lattice folding [79], which demonstrate conceptual feasibility but limited performance gains over classical baselines. Additional formulations map folding to Polynomial Unconstrained Binary Optimization (PUBO) formulation or Ising Hamiltonians and combine quantum subroutines with classical optimization to address scalability challenges [80]. Finally, Atari and Majd [81] propose a Quantum Genetic Algorithm for 2D HP folding, reporting faster convergence and improved accuracy for short to medium sequences.

#### Gene regulation and network biology

Gene Regulatory Network (GRN) inference and disease gene prioritization involve uncovering complex, nonlinear dependencies among thousands of genes and signaling molecules. Classical statistical and machine learning approaches, such as regression, correlation analysis, and Bayesian inference, often struggle to capture these combinatorial interactions. This challenge arises because the number of possible network states grows exponentially with the number of genes [6, 7], leading to prohibitive computational costs.

Quantum search-based approaches enable faster exploration of network configurations and more efficient detection of disease-relevant genes. Weidner et al. [27] introduce a quantum framework for Boolean GRNs analysis, using Grover’s search to identify predecessor states of attractors and Quantum Counting to estimate basin sizes, successfully reproducing classical attractor structures in simulation and limited hardware tests. Brisebois et al. [82] formulate protein co-regulatory network inference as a Boolean Satisfiability (B-SAT) problem and apply Grover’s algorithm to recover regulatory logic from sparse expression data, demonstrating robustness to noise on NISQ hardware. Variational circuit-based approaches are explored by Roman-Vicharra et al. [83], which use PQCs to infer gene interactions from single-cell RNA-seq data in simulation, and by Wang [84], which combines PQCs with Transformers to improve accuracy and training efficiency. Konar et al. [85] propose a variational quantum regression model for Alzheimer’s-related gene interaction inference, validated on a small simulated subsystem. Beyond GRNs, Saarinen et al. [86] present a quantum-inspired disease gene prioritization method based on continuous-time quantum walks on protein–protein interaction (PPI) networks, consistently outperforming classical diffusion-based rankings.

#### Sequence and motif analysis

Motif identification is particularly challenging, reducing to an NP-hard subgraph isomorphism problem [9], while protein and DNA similarity calculations, including k-mer–based comparisons, are limited by memory demands and high time complexity on large datasets [8]. As biological sequence data grow exponentially, these limitations highlight the need for alternative computational paradigms.

Several studies investigate quantum and quantum-inspired methods for sequence matching, similarity analysis, and Motif identification, mainly through theoretical and simulation-based evaluations. Miyamoto et al. [87] propose two quantum algorithms for Position Weight Matrix matching, one based on iterative scoring and another using Quantum Monte Carlo Integration (QMCI) [88], both rely on Quantum Amplitude Amplification [89] to achieve theoretical quadratic speedups, though the work remains purely theoretical. Balewski et al. [90] apply Grover’s search and quantum counting to DNA sequence similarity estimation using k-mer representations, demonstrating correct behavior and expected speedups on small synthetic datasets. Chagneau et al. [91] explore quantum-assisted protein similarity by combining quantum-generated reference sequences with quantum variants of classical alignment algorithms and a QAOA-solved conflict-graph QUBO, finding that the QAOA-based approach best matches BLASTP rankings in simulation. Ngo et al. [92] reformulate network Motif identification as a Hamiltonian optimization problem solved with QAOA, partitioning large networks to fit qubit limits and outperforming classical motif-finding tools on synthetic and real regulatory networks.

#### Transcriptomics and gene expression analysis

Gene expression analysis is computationally challenging because microarray and RNA-seq datasets contain tens of thousands of noisy, redundant, and often irrelevant genes, creating severe high-dimensional search spaces that classical methods struggle to handle.

Studies in this domain use quantum circuit-based search and measurement to capture complex dependencies in expression profiles, improving cancer gene prediction, biomarker identification, and viral-infection signature detection. Dabba et al. [93] propose a quantum-inspired extension of the Moth Flame Optimization (MFO) algorithm [94] for microarray feature selection, showing improved search exploration and superior SVM classification performance over classical MFO and other baselines. Swathi et al. [95] introduce Quantum Ant Lion Optimization, a qubit-inspired probabilistic feature-selection method that enhances exploration in noisy gene-expression data and achieves higher accuracy and precision-recall than classical and deep-learning approaches when paired with SVMs. Karthi et al. [96] present a hybrid framework combining classical preprocessing with a quantum dilated convolutional layer and GRU classifier for virus detection, reporting improved accuracy on COVID-19 and leukemia datasets.

#### Multi-omics data integration

Multi-omics integration creates datasets with tens of thousands of interacting features and nonlinear cross-omics dependencies that overwhelm classical clustering, classification, and feature-selection methods. The combinatorial explosion of possible feature subsets and cluster assignments further increases computational cost, limiting the accuracy of classical disease-subtyping and biomarker-discovery pipelines.

Recent studies show that quantum methods can improve feature-selection stability and capture subtle multi-omic patterns classical models miss. Saggi and Kais [97] propose a quantum neural network framework for lung adenocarcinoma classification using integrated DNA methylation, miRNA, and mRNA data, achieving strong predictive performance and identifying biologically plausible biomarkers. Quantum Fuzzy C-Means (QFCM) [98] extends classical FCM [99] by introducing quantum-based similarity measures for clustering protein sequences, producing more compact and well-separated clusters than classical methods. Mohammed and Ali [100] present a hybrid approach that combines classical K-means clustering with Quantum Cat Swarm Optimization for feature selection, improving SVM-based cancer subtype classification without directly encoding omics data into quantum states.

#### Protein design and engineering

Protein design and engineering remain computationally difficult because the space of possible amino acid sequences and corresponding 3D structures grows exponentially with protein length, making the problem NP-hard. As a result, classical approaches often become trapped in local minima.

Early studies demonstrate that quantum approaches allow simultaneous evaluation of many candidate configurations and help escape local minima, offering improved sampling of low-energy sequences compared to classical heuristics. Khatami et al. [29] formulate protein sequence design as a search problem over precomputed energy tables and apply Grover’s algorithm, evaluating simplified lattice and reduced HP models suitable for NISQ devices. Irbäck et al. [33] recast protein design under the 2D HP lattice model as a QUBO problem and solve it using quantum annealing, demonstrating that hybrid quantum-classical annealer reliably recover optimal sequences, although pure quantum annealer (pure QPU) remains limited to small instances due to noise and embedding constraints.

#### Phylogenomics and evolutionary analysis

Constructing phylogenetic trees is computationally challenging because the number of possible topologies grows super-exponentially with the number of species, making exhaustive search infeasible even for moderate datasets. Classical methods such as neighbor-joining, maximum likelihood, and Bayesian inference must heuristically explore this astronomically large space while also processing high-dimensional genetic distance matrices.

Normalized Minimum cut by Digital Annealer (NMcutDA) [101] is a quantum-inspired phylogenetic reconstruction method that formulates tree building as a normalized graph-cut problem optimized on a Digital Annealer. Protein sequences are converted into a similarity matrix using BLASTP and BLOSUM62 scores, which is then treated as a weighted graph. The bipartitioning step is encoded as a QUBO, and the annealer performs iterative bipartitions to construct the final tree.

### Thematic analysis

#### Confronting the noise and scalability hurdle

This theme examines how current studies address NISQ-device noise. As deep circuits on present-day hardware cannot yet solve full-scale biological problems, existing approaches rely on three common strategies: (i) explicit error-mitigation techniques, (ii) variational algorithms and shallow-circuit architectures, and (iii) systematic reduction of problem size.

**Explicit error-mitigation techniques:** Domingo et al. [60] introduce Data Re-Uploading Error Mitigation (DREM) strategy to improve robustness on NISQ devices, where shallow PQCs repeatedly “re-upload” classical features to refit the model around noise. Liliopoulos et al. [35] handle noise by using Pauli twirling [102], M3 readout correction [103], and dynamical decoupling [104], enabling hardware results to closely track ideal simulator outputs despite circuit depth. Varsamis et al. [45] reduce noise sensitivity at the circuit-design level by using compact basis encoding and extremely shallow compare-shift circuits. Bikku et al. [69] pursue an indirect strategy, using tensor-network compression and reinforcement-learning-based circuit optimization to achieve high qubit fidelity without dedicated mitigation routines.

**Variational algorithms and shallow circuits**: variational methods naturally tolerate noise when circuits remain shallow. Fang et al. [43] apply VQE, decomposing the overlap-graph Hamiltonian into 6–10-qubit subproblems to avoid deep, decoherence-prone circuits. Robert et al. [20] and Pamidimukkala et al. [31] reformulate folding as QUBO/Ising optimization and apply CVaR-VQE, which concentrates optimization on the low-energy tail of the distribution to reduce the influence of noisy measurements. Chandarana et al. [30] further enhance noise robustness by incorporating digitized counterdiabatic (CD) terms into their variational ansatz.

**Problem-size reduction**: the third group addresses NISQ limitations by reducing problem size. Akpinar et al. [52] compress microarray data from ~54 k features to a 15-qubit representation via normalization and PCA, enabling shallow variational classifiers despite potential information loss. Konar et al. [85] models Alzheimer’s gene regulation with eight qubits and symmetry constraints that halve entangling-gate demand while maintaining accuracy comparable to earlier GRN models [63]. Mensa et al. [28] apply PCA/ANOVA to reduce molecular fingerprints before QSVC classification [62], keeping circuits below ~24 qubits. Quantum Optimization for Motif Identification (QOMIC) [92] partitions large biological networks into smaller edge-subgraphs and runs QAOA on each compact Hamiltonian, merging results to recover motif structure without exponential qubit growth.

**Cost and limitations:** these strategies introduce clear trade-offs. Explicit Error-Mitigation techniques add circuit executions and calibration overhead, increasing runtime; variational and CD-enhanced ansatzes reduce depth but remain vulnerable to barren plateaus and high optimization cost; and problem-size reduction improves feasibility but may remove biologically meaningful dependencies, limiting analysis fidelity.

#### Quantum encoding

Encoding represents a foundational challenge in quantum bioinformatics. High-dimensional biological entities—such as DNA reads, protein structures, expression profiles, and interaction networks—must be transformed into qubit-compatible representations that preserve essential biological structure under the severe qubit and noise constraints of NISQ hardware. Table 3 categorizes the reviewed studies by encoding techniques.

**Table 3:** Publication by encoding technique.

**Encoding technique**	**Studies**
Hamiltonian (QUBO-based) encoding	[21, 22, 24, 25, 30, 32–34, 43, 67–69, 86, 92, 101]
Basis encoding	[23, 29, 45, 63, 70–72, 74, 81, 96, 100]
Angle (Rotation) encoding	[4, 28, 50, 51, 53, 56, 57, 65, 75, 77, 79, 83–85, 90, 93]
Amplitude encoding	[52, 59, 97, 98]
Hybrid encoding	[20, 26, 27, 31, 35, 44, 48, 49, 54, 58, 60, 61, 64, 80, 91]
Quantum-inspired	[46, 95]

**Hamiltonian (QUBO-based) encoding:** a prevailing trend is reformulating bioinformatics problems as QUBO or Ising Hamiltonians, where biological constraints become energy penalty terms so that the ground state corresponds to a biologically valid solution found by quantum annealers or variational eigensolvers.

In Genome-assembly tasks, Sarkar et al. [21] reformulate the OLC problem as a Binary Quadratic Model (BQM), where binary variables represent read-position decisions are mapped directly into a QUBO Hamiltonian. Boev et al. [22] similarly, map OLC assembly into QUBO form, encoding read placement along a Hamiltonian path through QUBO variables. Nałęcz-Charkiewicz et al. [24] transform DNA reads into cumulative-phase signals, compute pairwise similarities via Pearson correlation, and formulate genome assembly as a TSP-QUBO in which read overlaps and constraints are encoded as quadratic Hamiltonian penalties. Fang et al. [43] also express hybrid *de novo* assembly as a QUBO and solve it with VQE. Chen et al. [32] formulate haplotype assembly as a QUBO whose binary variables form the QUBO matrix entries, which capture read overlap consistency scores, conflict penalties, and linkage relationships between reads.

In protein modeling, Irbäck et al. [25] construct a QUBO formulation of the HP lattice model for protein folding, encoding steric constraints and interaction energies directly into the Hamiltonian. Chandarana et al. [30] similarly translate protein geometry, turn constraints, chirality, and Miyazawa–Jernigan interaction energies into an Ising Hamiltonian. Irbäck et al. [33] formulate HP-model sequence optimization as a QUBO/Ising Hamiltonian.

In drug-discovery and molecular-interaction tasks, Ding et al. [67] formulate molecular docking as a maximum-vertex-weight-clique QUBO [105]. Ligand–receptor interaction variables are mapped into a binary QUBO and converted into a cost Hamiltonian. Similarly, Lancellotti et al. [34] cast the docking problem as a weighted subgraph-isomorphism problem and embed all structural and geometric constraints into a QUBO/Ising Hamiltonian. Other works embed molecular electronic-structure Hamiltonians into variational circuits: Surya Prakash et al. [68] and Bikku et al. [69] use VQE to estimate ground-state energies serving as quantum-derived features for downstream prediction.

In network-level biological tasks, Saarinen et al. [86] encode PPI networks by using their adjacency matrices as Hamiltonians for continuous-time quantum walks. QOMIC [92] reformulates Motif identification as an unconstrained integer optimization whose objective and combinatorial constraints map into an Ising Hamiltonian.

In phylogenetics, Onodera et al. [101] reformulate the phylogenetic normalized-cut (Ncut) partitioning task as a QUBO/Ising problem, where each sequence is assigned a binary decision variable indicating cluster membership.

**Basis encoding:** basis encoding is among the most direct strategies, mapping biological objects onto computational-basis states.

In protein structure prediction tasks, Mao et al. [74] map 388 possible protein conformations to computational basis states and model protein folding as a quantum walk over this basis-encoded landscape. Wong and Chang [70] encode hydrophobicity, lattice-transition directions, 3D coordinates, and adjacency indicators directly as basis qubits; their follow-up work [71] similarly encodes HP residues and lattice transitions for parallel energy evaluation. Casares et al. [23] represents a protein’s torsion-angle configuration space as binary strings, letting a Szegedy-style quantum Metropolis algorithm to operate entirely over basis-encoded angle states. Atari and Majd [81] encode the HP-model four directions as 2-bit qubit patterns within a Quantum Genetic Algorithm. Bhuvaneswari et al. [72] apply basis encoding of hydrophobicity and lattice-transition directions for minimal-energy conformations.

In sequence-oriented tasks, Varsamis et al. [45] map nucleotides to 2-qubit binary states, a representation aligned with gate-based operations such as quantum comparison and shifting. Khatami et al. [29] encode candidate protein sequences by assigning each amino acid a binary string and each designable site its own qubit register. Dorsey et al. [63] similarly, basis-encode compressed molecular fingerprints, mapping each bit to computational-basis states.

Basis encoding also appears in feature-selection frameworks. Mohammed and Ali [100] represent each candidate feature subset as a multi-qubit bitstring, using superposition and entanglement to explore update strategies in parallel before collapsing to a final selection. Karthi et al. [96] map gene-expression vectors into basis states via an encoding operator.

**Angle encoding:** angle encoding is a common NISQ-compatible technique that maps continuous biological or clinical features into quantum states through single-qubit rotations.

In drug-discovery and molecular-modeling tasks, Mensa et al. [28] employ a ZZFeatureMap in which each molecular descriptor becomes a rotation parameter. Ganguly et al. [65] apply pure Rx rotation to normalized molecular features.

In QML, Kunjachena and Kavitha [50] encode clinical attributes as Ry rotations, letting each numerical feature modulate a quantum rotation in the Quantum GRU layer. Magendiran et al. [51] encode normalized gene-expression features using Ry/Rz rotations on a 4-qubit quantum dilated convolution module, with one qubit per value in a 2 × 2 feature patch. Hong et al. [77] map every pixel in protein 2D feature maps to Ry rotations. The encoded qubits serve as the input to a variational quantum convolutional filter.

Angle encoding is also used in trainable embedding frameworks, where rotation gates serve as learnable parameters. Li et al. [4] initialize circuits with tunable Rz/Ry rotations and refine them through variational layers with Rzz entanglers. Choppara and Lokesh [57] encode viral-mutation features by mapping each input value to Ry/Rz rotations within a QLSTM cell. These input-dependent rotations and trainable variational parameters enable adaptive quantum state evolution throughout the recurrent structure. Nguyen [53] similarly, loads clinical or molecular descriptors through tunable rotation gates (Rx/Ry/Rz) inside a variational QNN ansatz. Kundu et al. [56] assign trainable rotation parameters to amino acids within a QNLP-inspired feature map, producing learnable quantum embeddings. Roman-Vicharra et al. [83], Wang [84], and Konar et al. [85] encode gene-expression levels and classical activation ratios via Ry rotations and represent regulatory interactions through controlled-Ry gates. The rotation parameters are trained to align the quantum output distribution with observed single-cell activation patterns. Muscalagiu [79] encodes protein-folding states using Rz rotation, where a classical preprocessing layer applies trainable weights.

In structural bioinformatics, Varsamis and Karafyllidis [75] encode protein dihedral angles ($\varphi, \psi$) as phase-shift parameters in a quantum-walk model. Balewski et al. [90] use parallel uniformly controlled rotations (pUCRs) to encode DNA k-mers as Pauli-Y rotations (0 or π). In feature-selection settings, Dabba et al. [93] represents each gene as a rotation-parameterized qubit, enabling probabilistic feature selection through angle updates that balance exploration and exploitation.

**Amplitude encoding:** amplitude encoding compactly embeds high-dimensional biological data into logarithmic qubit space by mapping normalized feature vectors to quantum-state probability amplitudes.

Dong et al. [59] use L2-normalized feature values as probability amplitudes in the computational basis. QFCM [98] represents each normalized protein sequence as amplitudes of an N-qubit state. Saggi and Kais [97] encode 16- and 64-dimensional multi-omic cancer features into 4- and 6-qubit amplitude states. Finally, Akpinar et al. [52] employ amplitude encoding for a high-dimensional gene-expression vector and transform ~54 k normalized gene-expression features into 15-qubit amplitude states after dimension reduction.

**Hybrid encoding:** hybrid encoding strategies combine multiple quantum data-representation methods to capture different biological features.

Robert et al. [20] combine Hamiltonian and basis encoding for protein folding. Each conformation is represented as a computational basis bitstring, while lattice geometry, steric exclusion, chirality, and pairwise interaction are encoded as Pauli-string energy terms. Pamidimukkala et al. [31] encode the protein-folding problem using a turn-based binary representation, where each lattice move uses six binary qubits and the resulting configurations form a QUBO subsequently mapped to an Ising Hamiltonian. Wang and Zhou [80] basis-encode tetrahedral turns, formulate folding as a PUBO, and convert it to a local Ising Hamiltonian optimized with an angle-encoded variational ansatz. Varsamis et al. [44] basis-encode each read as a computational-basis state in the quantum walk’s position register, while edge weights and overlap constraints are encoded through a QUBO/Ising Hamiltonian. Angle-based phase shifts incorporate edge potentials during the quantum walk. Chagneau et al. [91] basis-encode the 20 amino acids, then construct amplitude-encoded quantum states whose amplitudes reflect empirical amino-acid occurrence probabilities, prepared via multi-controlled Ry rotations.

Li and Ghosh [58] apply a dual-encoding strategy: the encoder uses amplitude encoding to load molecular data into probability amplitudes, while the decoder and scalable variant use angle encoding for hardware-efficient latent reconstruction. Lau et al. [26] combine basis encoding for binary mutation-ligand descriptors with Rx-based angle encoding for continuous biochemical features. Variational training further tunes rotation parameters. Domingo et al. [60] embed CNN-derived features using amplitude encoding, supplemented by Hybrid Angle Encoding (HAE) that maps feature blocks through rotation gates. Amplitude encoding yields superior affinity-prediction performance. Choppara et al. [64] use a rotation-based quantum feature map where compound-protein features are Ry-encoded and entangled to create nonlinear representations. Avramouli et al. [61] compare angle, dense-angle, and amplitude encoding for transforming 256 biochemical features into 8-qubit states, pairing each with variational evolution blocks. Liliopoulos et al. [35] combine basis encoding for Grover-based docking-site search with amplitude encoding for SWAP-test similarity evaluation, integrating discrete search and continuous scoring in one hybrid workflow.

GenÇ [48] applies angle encoding to diabetes-indicator features by mapping each value to an Ry rotation before passing through a quantum feature map optimized via the COBYLA algorithm [106]. Ullah et al. [49] use amplitude encoding to embed clinical and lifestyle features into log₂(N) qubits, enriched by a parameterized quantum feature map. Astuti et al. [54] adopt a hybrid trainable feature-map approach in which transcriptomic features pass through a classical layer before Rz-rotation encoding. A ZZFeatureMap is also used for QSVC, making the overall pipeline an adaptive form of angle-based quantum feature mapping. Weidner et al. [27] encode Boolean network states directly in basis form and incorporate angle-encoded Ry rotations to bias initial gene activities, combining discrete basis representation with continuous rotation-based tuning.

**Quantum-inspired (no specific quantum encoding scheme):** Ahmad et al. [46] do not perform any quantum data encoding, operating entirely within a quantum-inspired classical framework. CNN-derived leukocyte features are ranked using a quantum-inspired algorithm that mimics superposition and amplitudes through classical coefficients. Swathi et al. [95] similarly, avoid quantum state preparation, instead using quantum-inspired stochastic operators in the Quantum Ant Lion Optimization algorithm to enhance feature selection.

## Discussion and conclusion

Quantum computing shows strong potential for addressing computationally intensive problems in bioinformatics, which has driven growing research activity in quantum bioinformatics. The reviewed studies indicate a clear upward trend, reflecting a rapidly expanding yet still early-stage field. Current research is largely concentrated in drug discovery, protein structure prediction, and machine-learning-based tasks, where data complexity and computational cost are high. Quantum machine learning methods dominate the landscape, and many studies combine quantum computing with artificial intelligence to improve efficiency and model complex biological data.

Despite this progress, most studies rely on classical simulation, indicating that current efforts focus primarily on feasibility rather than demonstrating hardware-level advantage. A gradual shift toward real quantum hardware is evident, but experiments remain limited in scale due to noise, restricted qubit counts, and shallow circuit depth. To address these limitations, researchers commonly adopt hybrid quantum–classical approaches, variational algorithms, error mitigation strategies, and problem-size reduction. Data encoding remains a fundamental challenge, as biological data are highly complex and must be mapped onto limited and noise-sensitive qubits, often requiring trade-offs between expressivity and feasibility.

Across the literature, common methodological patterns emerge. Many approaches reformulate bioinformatics problems into discrete optimization frameworks, such as QUBO or Ising models, which require simplifying biological systems through reduced feature spaces or abstract representations. These simplifications, along with reliance on near-ideal simulation environments and extensive classical preprocessing, limit biological realism and practical applicability. As a result, most current methods are designed to operate within NISQ constraints rather than fully capture the complexity of real-world biological systems.

The overall maturity of the field remains exploratory. Most studies demonstrate feasibility on small or synthetic datasets, with limited validation on large-scale or real biological data. Scalability is a major challenge, as many formulations grow exponentially with problem size, restricting their applicability. While hybrid approaches partially alleviate these issues, they do not yet offer clear scalability advantages over classical methods.

Finally, it is important to distinguish between theoretical proposals, simulation-based studies, and hardware implementations. As summarized in Table 2 in the supplemental document, a large portion of the literature remains theoretical or simulator-based, where hardware constraints are not fully represented. Only a small number of studies reports experiments on real quantum devices, and these are limited to small-scale problems. This gap highlights the difference between conceptual potential and practical deployment, suggesting that many reported advantages remain conditional on idealized settings. Overall, quantum bioinformatics shows promise, but significant challenges remain before achieving practical and scalable real-world impact.

**Key Points**

- **Promise and impact:** quantum computing holds significant promise for accelerating and enhancing the analysis of complex problems in computational biology.
- **Early-stage growth:** quantum bioinformatics shows increasing research activity and interest, but both QC technologies and their integration with biological applications remain at a nascent stage.
- **Simulation-driven research:** most studies rely on classical simulations of quantum algorithms, prioritizing feasibility and proof-of-concept demonstrations over clear hardware advantage, with only limited progression toward real-device implementations.
- **Fundamental technical limitations:** hardware noise, limited qubit availability, encoding difficulty, and scalability constraints restrict biological realism and methodological robustness.
- **Need for further development:** real-world applications will require advances in hardware capacity and improved methods for modeling complex biomolecular systems.

## Supplementary Material

Scopping_and_Mapping_Review-Supplemental_Document_bbag383

## Data Availability

The data underlying this article are available in the article and its supplemental document.
