# Supplementary material for: Quantum bioinformatics: a systematic review of methods, trends, and challenges
Source: Brief Bioinform. 2026 Jul 17;27(4):bbag383. doi: 10.1093/bib/bbag383 (PMC13379070; doi:10.1093/bib/bbag383)
Supplement: Scopping_and_Mapping_Review-Supplemental_Document_bbag383 [file scopping_and_mapping_review-supplemental_document_bbag383.docx]

**A Systematic Review Mapping on**

**Quantum Bioinformatics**

**Supplemental Documentation**

# Search Strategy

The following is the exact search string for the Scopus database, so that the search can be replicated by readers. This search strategy was adapted to Web of Science and Google Scholar based on the syntax used in each source.

( TITLE (( "quantum" OR "quantum computing" OR "quantum algorithm" OR "Grover's algorithm" OR "qaoa" OR "quantum optimization" OR "quantum walk" ) AND ( "genomics" OR "DNA sequence" OR "RNA sequence" OR "genome assembly" OR "haplotype phasing" OR "variant calling" OR "phylogenetic tree" OR "evolutionary analysis" OR "comparative genomics" OR "DNA sequencing" OR "genome" OR "gene" OR "motif" OR "motif prediction" OR "DNA similarity" OR "sequence assembly" OR "position weight matrix")) OR TITLE (("quantum" OR "quantum machine learning" OR "QML" OR "quantum neural network" OR "VQE" OR "quantum optimization" OR "QAOA" OR "QGNN" OR "QNN") AND ("protein" OR "protein folding" OR "protein structure" OR "proteomics" OR "PPI" OR "protein-protein" OR "protein-protein interaction" OR "protein-ligand interaction" OR "protein-ligand" OR "protein-DNA interactions" OR "protein structure prediction")) OR TITLE (("quantum" OR "quantum simulation" OR "VQE" OR "QAOA" OR "variational quantum algorithm") AND ("drug discovery" OR "drug resistance" OR "compound screening" OR "interaction energy" OR "personalized medicine" OR "drug design" OR "biomarker")) OR TITLE (("quantum" OR "quantum computing" OR "quantum optimization" OR "quantum clustering" OR "quantum machine learning" OR "QML" OR "quantum kernel" OR "QAOA") AND ("metagenomics" OR "microbiome" OR "microbial community" OR "taxonomic classification")) OR TITLE (("quantum" OR "quantum simulation" OR "quantum Hamiltonian" OR "quantum coherence" OR "quantum tunneling" OR "quantum modeling") AND ("enzyme kinetics" OR "signaling pathways" OR "photosynthesis" OR "olfaction" OR "ion channels" OR "metabolic pathway" OR "biological process")) OR TITLE (("quantum" OR "quantum machine learning" OR "QML" OR "quantum classifier" OR "quantum neural network" OR "quantum SVM" OR "QSVM" OR "quantum PCA") AND ("bioinformatics" OR "multi-omics" OR "gene expression" OR "single-cell analysis" OR "transcriptomics" OR "epigenomics" OR "dimensionality reduction" OR "biostatistics" OR "bioinformatics" OR "pattern matching")) OR TITLE (("quantum" OR "quantum computing" OR "quantum prediction" OR "QML" OR "quantum machine learning" OR "quantum simulation" OR "quantum epitope mapping") AND ("epitope" OR "antigen" OR "vaccine design" OR "immune response" OR "antigen-antibody interaction" OR "immunogenicity" OR "Immunolog*" OR "vaccine")) OR TITLE (("quantum" OR "quantum computing" OR "quantum classification" OR "quantum sequence analysis" OR "QML" OR "quantum machine learning" OR "quantum alignment") AND ("virology" OR "viral genome" OR "virus" OR "virus mutation" OR "viral mutation" OR "host-pathogen interaction" OR "epidemiology" OR "SARS-CoV-2" OR "variant tracking")) OR TITLE (("quantum" OR "quantum optimization" OR "QAOA" OR "quantum annealing" OR "QUBO" OR "quantum graph") AND ("sequence alignment" OR "bioinformatics" OR "gene regulatory network" OR "biomarker discovery" OR "systems biology" OR "PPI network" OR "metabolic network" OR "Protein-Protein interaction networks" OR "biomarker")) OR TITLE (("quantum" OR "quantum data encoding" OR "qubit encoding" OR "quantum circuit design" OR "quantum error mitigation" OR "NISQ" OR "quantum hardware-aware") AND ("biological data" OR "sequence encoding" OR "bioinformatics" OR "bioinformatics algorithm" OR "computational biology")) AND NOT TITLE ("quantum dot*" OR "quantum chemical*" )) AND PUBYEAR > 2014 AND PUBYEAR < 2026 AND ( LIMIT-TO ( SUBJAREA , "BIOC" ) OR LIMIT-TO ( SUBJAREA , "PHYS" ) OR LIMIT-TO ( SUBJAREA , "COMP" ) OR LIMIT-TO ( SUBJAREA , "PHAR" ) OR LIMIT-TO ( SUBJAREA , "MATH" ) OR LIMIT-TO ( SUBJAREA , "MEDI" ) OR LIMIT-TO ( SUBJAREA , "MULT" ) OR LIMIT-TO ( SUBJAREA , "AGRI" ) OR LIMIT-TO ( SUBJAREA , "IMMU" ) ) AND ( LIMIT-TO ( DOCTYPE , "cp" ) OR LIMIT-TO ( DOCTYPE , "ar" ) ) AND ( LIMIT-TO ( LANGUAGE , "English" ) )

# Descriptive Analysis

Table 1. Publication by Bioinformatics Domains

| **Bioinformatics Domain** | **Studies** |
| --- | --- |
| Drug Discovery and Molecular Interaction Modeling | [4], [23], [24], [25], [26], [27], [28], [29], [30], [31], [32], [33], [34], [35], [36] |
| Proteomics and Protein Structure Prediction | [37], [38], [39], [40], [41], [42], [43], [44], [45], [46], [47], [48], [49], [50] |
| Classification and Machine Learning in Bioinformatics | [51], [52], [53], [54], [55], [56], [57], [58], [59], [60], [61] |
| Genomics and Genome Assembly | [62], [63], [64], [65], [66], [67], [68] |
| Gene Regulation and Network Biology | [69], [70], [71], [72], [73] |
| Sequence and Motif Analysis | [74], [75], [76], [77] |
| Multi-Omics Data Integration | [78], [79], [80] |
| Transcriptomics and Gene Expression Analysis | [81], [82], [83] |
| Protein Design and Engineering | [84], [85] |
| Phylogenomic and Evolutionary Analysis | [86] |

# Qualitative Analysis

## Genomics and Genome Assembly

The authors of QuASeR [1] introduce an end-to-end quantum formulation of *de novo* genome assembly that recasts the Overlap-Layout-Consensus (OLC) problem as a QUBO model for both quantum annealing and gate-based (QAOA). The results show that quantum annealing achieves optimal read ordering, while QAOA yields directionally consistent but approximate solutions.

Boev et al. [2] present a proof-of-concept application of quantum annealing and quantum-inspired digital annealing to genome assembly via QUBO reformulation. Their results show that small synthetic sequences can be assembled on existing hardware, whereas scalability is constrained by qubit connectivity and noise.

Nałęcz-Charkiewicz et al. [3] propose a hybrid quantum-classical approach to *de novo* DNA assembly by reformulating the overlap-based task as a Traveling Salesman Problem (TSP) and expressing it as a QUBO solved using a quantum annealer. The method is tested on both synthetic and real genomes and performs well on smaller datasets.

Fang et al. [4] propose a divide-and-conquer hybrid quantum algorithm that reformulates genome assembly as an optimization problem in QUBO form and solves subproblems using VQE with a problem-inspired ansatz. Classical preprocessing breaks the assembly graph into smaller modules, enabling execution on small qubit systems and larger simulated Hamiltonians.

Varsamis et al. [5] introduce a hybrid quantum-classical framework for genome assembly that integrates QAOA-based Max-Cut, quantum walks, and Hamiltonian path solvers. The method decomposes the overlap graph into smaller subgraphs for quantum processing. It uses quantum walks to explore dense regions and Ising-formulated Hamiltonian path solvers for higher-level structure. The study demonstrates feasibility on synthetic data but remains simulation-only and untested for scalability.

Chen et al. [6] present a hybrid quantum-classical approach to haplotype-resolved genome assembly by expressing the problem as a QUBO suitable for quantum annealers. Classical Google OR-Tools handle large-scale optimization, while quantum annealing refines smaller subproblems. The pipeline performs well on synthetic datasets and local segments of real genomes.

Varsamis et al. [7] introduce a gate-based algorithm for reference-guided DNA alignment, reformulating the alignment process into quantum “compare” and “shift” operations and exploiting superposition for parallel matching. Simulations on small synthetic sequences confirm correct alignments and linear qubit scaling.

## Classification and Machine Learning in Bioinformatics

The authors of [8] introduce a quantum-inspired deep learning pipeline for white blood cell classification that combines transfer learning-based feature extraction with a quantum-inspired evolutionary algorithm for feature reduction. The method improves search efficiency over classical algorithms and shows the potential of quantum-inspired optimization despite using only classical hardware.

Darolia et al. [9] present a hybrid framework for predicting cardiovascular disease. It integrates classical deep learning with Quantum Neural Network (QNN) elements, using a self-improved Aquila Optimization algorithm [10] to select informative clinical features. A combined LSTM-QNN classifier processes the optimized features, showing improved predictive performance over classical baselines.

GenÇ [11] compares classical and quantum ML methods for diabetes prediction using a reduced feature set. The study implements Quantum Support Vector Machine (QSVM) on simulators, showing that quantum models can achieve performance comparable to classical approaches while offering a compact qubit-based representation.

Ullah et al. [12] evaluate two QML models for sarcopenia risk prediction: a Quantum K-Nearest Neighbor classifier (QKNN) using fidelity-based similarity measures and an Amplitude-Encoding Variational Quantum Classifier (AE-VQC) combining variational circuits with classical optimization. Although limited to simulated circuits and small datasets, both models demonstrate competitive performance on reduced feature sets compared with classical baselines.

Kunjachena and Kavitha [13] introduce a hybrid quantum-classical approach that integrates an optimization-based feature selector with a quantum attention-enhanced BiGRU classifier for heart-disease prediction. Evaluated on multiple clinical datasets using simulated circuits, the model outperforms classical baselines.

N et al. [14] present a modified version of Quantum Dilated Convolutional Neural Network (QDCNN) [15] for cancer prediction using gene-expression data. The framework combines classical preprocessing, feature fusion, and QDCNN to improve nonlinear representation and reduce computational cost. Tested on simulators, the approach yields higher accuracy and efficiency than several deep-learning baselines.

Akpinar et al. [16] propose a Deep Variational Quantum Classifier for brain tumor classification using high-dimensional microarray data. After classical preprocessing, PCA, and encoding the gene-expression features into quantum states, the model is evaluated entirely on simulated circuits.

Nguyen [17] introduces a QNN framework for biomarker discovery, using multi-omics datasets from Cancer Genome Atlas [18]. The model formulates biomarker identification as a quantum feature-selection task and shows that QNNs can explore large combinatorial spaces to identify biologically plausible candidates. However, the results are based only on simulations.

Astuti et al. [19] evaluate a hybrid quantum-classical approach for biomarker classification in renal cancer, combining Neural Quantum Embedding [20] with QSVC and QNN models. After preprocessing and dimensionality reduction, quantum feature maps outperform classical baselines on smaller, balanced datasets, but results are limited to simulations and affected by data imbalance.

Kundu et al. [21] explore Quantum Tensor Networks (QTNs) [22] for protein-sequence classification using two architectures, Path Tensor Network and Convolutional Tensor Network. Simulated on UniProt [23] sequences, the models achieve competitive accuracy with far fewer parameters than classical deep-learning counterparts.

Choppara and Lokesh [24] propose a Quantum LSTM model for SARS-CoV-2 mutation prediction, integrating quantum operations into a recurrent architecture to model nonlinear genomic dependencies. Tested on small viral sequence datasets, the model outperforms classical baselines in simulation.

## Drug Discovery and Molecular Interaction Modeling

The authors of [25] propose a hybrid Quantum Generative Adversarial Network (QGAN) for small-molecule generation, integrating parameterized quantum circuits (PQCs) with classical neural networks to explore chemical space in drug discovery more efficiently. The model achieves performance comparable to classical GANs [26] with fewer parameters, though it is tested only on simulated circuits and constrained by shallow NISQ architectures.

Li and Ghosh [27] develop scalable quantum autoencoder architectures for molecular representation learning using Variational Quantum Circuits and QNN. The approach shows faster convergence and competitive reconstruction accuracy on lower-dimensional inputs, with the scalable variant extending to larger molecular structures.

Mensa et al. [28] investigate a hybrid quantum-classical approach for ligand-based virtual screening using Quantum Support Vector Classifiers (QSVCs) [29] with quantum kernel estimation. After heavy feature reduction to meet qubit limits, QSVCs outperform several classical baselines on multiple datasets but remain constrained by small qubit counts.

The study by Dorsey et al. [30] evaluates quantum models for predicting antimalarial bioactivity using compressed molecular features. Two classifiers, a Variational Quantum Classifier and a Quantum Fourier Transform Classifier (QFTC), are tested on simulated circuits, with the latter showing improved performance but still lagging classical models trained on full features.

Lau et al. [31] develop a hybrid quantum-classical workflow for predicting how protein mutations affect ligand binding, integrating QNNs and Variational Quantum Circuits into traditional computer-aided drug design (CADD) pipelines. The approach shows comparable performance to classical methods on SARS-CoV-2 protease inhibitors across simulated and hardware evaluations.

Dong et al. [32] introduce hybrid deep-learning architectures that embed PQCs within classical convolutional neural networks (CNN), graph convolution networks [33], and graph isomorphic networks [34] models for binding-affinity prediction. Across benchmarks, the hybrid models achieve competitive or superior performance with fewer parameters, though results are largely simulation-based.

Domingo et al. [35] present a hybrid fusion model for protein-ligand binding affinity prediction that combines 3D-CNN and graph-based molecular representations with a QNN component, and then PQCs are incorporated into the fusion layer. Although executing on simulators, the hybrid approach outperforms the classical fusion baseline and demonstrates smoother convergence.

Avramouli et al. [36] integrate classical CNN-based feature extraction with Variational Quantum Regression layers for binding-affinity prediction. The study reports improved error metrics and faster convergence relative to classical fully connected networks, while also reducing trainable parameter counts. However, evaluation is limited to simulated circuits.

Choppara et al. [37] evaluate a QSVM for compound-protein interaction (CPI) prediction using curated datasets. The QSVM consistently outperforms classical baselines and demonstrates the ability of quantum kernels to capture nonlinear molecular-protein relationships. The study is limited to simulated circuits and modest dataset sizes.

Ganguly et al. [38] propose a hybrid Quantum Graph Neural Network (QGNN) that integrates graph convolution layers with variational quantum circuits for protein-ligand interaction prediction. Although relying on simulation, the hybrid model surpasses classical GNN performance and converges more efficiently.

The study by Liliopoulos et al. [39] proposes a fully quantum method for identifying protein-ligand docking sites by framing docking as a quantum search and similarity-matching problem. The approach encodes interaction patterns as quantum states and uses Grover’s search and SWAP-test [40] similarity measures. Implemented on simulators and hardware, the method identifies docking sites and successfully localizes interaction regions.

Lancellotti et al. [41] present a proof-of-concept quantum framework for molecular docking by formulating the problem as a QUBO instance. Using simplified ligand-protein representations suitable for NISQ constraints, the study shows that QA delivers higher-quality solutions and faster execution than both QAOA and classical baselines, while QAOA remains limited by shallow circuit depth and noise.

Ding et al. [42] reformulate molecular docking as a Maximum Vertex Weight Clique problem and solve it using QAOA and its improved variant, Digitized-Counterdiabatic QAOA (DCQAOA) [43]. Demonstrated on simplified molecular representations and pharmacophore-based interaction graphs, the approach shows that DCQAOA converges more efficiently and accurately than standard QAOA in simulation.

Surya Prakash et al. [44] couples VQE-derived quantum features with classical Graph Neural Networks for molecular property prediction. VQE provides quantum estimates of molecular interactions, while the GNN interprets the molecular graph structure to learn complex chemical relationships and molecular properties. In simulation, the model outperforms classical baselines, suggesting the potential benefits of quantum feature extraction.

Bikku et al. [45] introduce a QNN architecture that incorporates reinforcement-learning optimization, tensor-network scalability, and noise-mitigation techniques for drug-discovery applications. Although simulation-based, the model shows strong predictive performance and robustness relative to classical and earlier quantum baselines such as QSVM and quantum decision trees.

## Proteomics and Protein Structure Prediction

Mao et al. [46] present a quantum-inspired framework for protein folding and misfolding by modeling conformational transitions as a quantum walk over a lattice-derived state graph. Using Lindblad equations [47] to incorporate environmental dissipation, the study analyzes folding pathways and identifies regime shifts between correctly folded and misfolded states. Although implemented classically, the work provides a theoretical foundation.

Wong and Chang [48] propose an algorithm for PSP based on the hydrophobic-hydrophilic (HP) model. It uses Grover’s search to explore conformational space in superposition and identify low-energy structures. Simulated experiments on small sequences validate the algorithm’s behavior and expected quadratic speedup.

Hong et al. [49] apply Quantum Convolutional Neural Networks (QCNNs) to protein distance prediction, replacing classical convolution layers with convolutional filters. QCNN implemented via variational quantum circuits, achieved accuracy comparable to state-of-the-art CNNs, but with faster convergence and fewer training epochs.

Robert et al. [50] introduce a hybrid algorithm for protein folding. It formulates the task as a combinatorial optimization problem solvable with Grover’s search. Optimized through a Conditional Value-at-Risk Variational Quantum Eigensolver (CVaR-VQE) [51] with a genetic algorithm, the approach was tested on synthetic lattice models of short peptides. The approach demonstrates consistent convergence toward low-energy conformations and requires relatively few qubits, with small-scale tests also run on hardware.

The study proposed by Atari and Majd [52] presents a Quantum Genetic Algorithm (QGA) for solving the 2D hydrophobic-polar protein-folding problem. The QGA achieves faster convergence and improved folding accuracy for short to medium-length sequences. Although the advantage diminishes for longer sequences, the QGA consistently outperforms classical GA in computational efficiency.

QFold [53] is a hybrid quantum-classical framework to address the non-lattice protein folding problem. Folding is formulated as an optimization over continuous torsion angles, with Minifold [54] predicting an initial conformation and a quantum-walk-based Metropolis [55] procedure that iteratively searches for low-energy states. While applied only to short peptides and reliant on precomputed energy tables, the method demonstrates polynomial speedups over classical Metropolis sampling [56] in simulated tests.

Wong and Chang [57] develop a Grover-based quantum algorithm for PSP under the 2D HP model, using quantum superposition to evaluate all conformations in parallel and identify low-energy folds. Although the study is limited to simplified models and small toy examples, simulated tests on small sequences confirm the algorithm’s expected quadratic speedup over exhaustive classical search.

Irbäck et al. [58] reformulate the HP lattice model protein folding as a QUBO problem equivalent to an Ising Hamiltonian and apply quantum annealing to explore low-energy configurations. The workflow performs well on short and medium-length benchmark sequences, while pure QPU execution is restricted to very small proteins due to hardware noise. The study finds quantum annealing to be superior to classical simulated annealing [59].

Muscalagiu [60] applies Quantum Reinforcement Learning [61] to the protein-folding problem by replacing the neural network in Deep Q-Learning with a PQC, forming a QNN that approximates Q-values. Evaluated on 2D and 3D HP lattice models, the approach identifies near-optimal low-energy folds for several sequences, demonstrating conceptual feasibility. However, classical baselines converge faster and more reliably.

Varsamis and Karafyllidis [62] propose a hybrid quantum-classical algorithm for protein folding prediction. The method employs quantum walks to model the peptide backbone as a one-dimensional lattice. Each amino-acid side chain effects are incorporated through a cost function, and the system is iteratively optimized via a classical Metropolis algorithm [56]. Although tested only on a short peptide and small toy systems, the algorithm consistently converges to biologically plausible conformations.

Pamidimukkala et al. [63] introduce a gate-based quantum framework for PSP by formulating the HP folding problem as a QUBO and solving it with a VQE-CVaR optimizer. Implemented on hardware for short sequences, the method produced low-energy folds comparable to classical baselines such as simulated annealing and CPLEX [64]. Performance declines on larger sequences due to noise and circuit limitations.

Wang and Zhou [65] map lattice protein folding to a Polynomial Unconstrained Binary Optimization (PUBO) formulation and a local Ising Hamiltonian. By combining quantum phase estimation with classical gradient optimization, the method aims to mitigate scalability issues due to exponentially small spectral gaps and barren plateaus [66], [67]. Tested in simulation on tetrahedral lattice proteins, the approach achieves polynomial gate complexity.

The study by Bhuvaneswari et al. [68] applies Grover’s search to PSP under the 3D HP model. It uses amplitude amplification to identify low-energy conformations within a global superposition of folds. Simulated results show improved fold prediction accuracy and indicate potential quadratic speedups over classical search. The predicted structures are also used to enhance protein-protein interaction (PPI) classification relative to classical baselines.

Hegade et al. [69] propose a hybrid quantum-classical folding algorithm that employs Digitized Counterdiabatic protocols to reduce circuit depth and improve optimization stability. The folding task is modeled on a 3D tetrahedral lattice, and the Miyazawa-Jernigan [70] interaction mode. The model achieves faster convergence and higher success rates than QAOA-style circuits and is validated on hardware devices for small peptides.

## Gene Regulation and Network Biology

Weidner et al. [71] present a quantum framework for simulating gene regulatory networks (GRNs) modeled as Boolean systems. The method applies Grover’s search to identify predecessor states leading to known attractors and Quantum Counting [72] to estimate basin sizes. Applied to small GRN models, the method reproduced classical attractor structures in both simulation and limited hardware tests, despite noise-related deviations. Brisebois et al. [73] continued their work by formulating protein co-regulatory network inference as a Boolean Satisfiability (B-SAT) problem using sparse protein expression data. They encode the regulatory logic of a five-protein network as Boolean constraints and solve the resulting combinatorial search using Grover’s algorithm. The method was evaluated on both quantum simulators and real NISQ hardware, successfully recovering the true regulatory logic despite hardware noise.

qscGRN [74] uses PQCs to infer gene regulatory interactions from single-cell RNA-seq data. The task is to predict the regulatory interactions between genes, together with the strength of these interactions. Encoding each gene as a qubit, the model is trained to match predicted and observed gene-activation distributions and successfully recovers known interactions. The study is implemented only in simulation and is limited to a six-gene system.

Q-TetoFormer [75] combines a PQC with a Transformer to enhance GRN prediction from single-cell RNA-seq data. The quantum circuit provides initial gene-interaction representations, which are refined through attention mechanisms, yielding notable improvements over earlier models such as qscGRN in both accuracy and training efficiency.

Konar et al. [76] propose Alz-QNet, a variational quantum regression model for inferring gene interactions in Alzheimer’s disease from single-nucleus RNA-seq data. Each gene is encoded as a qubit, and a symmetry constraint is used to reduce entanglement overhead. The model recovers biologically consistent regulatory relationships in an eight-gene subsystem but is evaluated only in simulation.

Saarinen et al. [77] introduce a quantum-inspired disease gene prioritization method based on continuous-time quantum walks [78] applied to PPI networks. By modeling the adjacency matrix as a Hamiltonian, the method captures richer diffusion behavior than classical walk-based approaches and consistently improves ranking metrics across multiple disease datasets.

## Sequence and Motif Analysis

Miyamoto et al. [79] propose two quantum algorithms for Position Weight Matrix matching, one based on iterative scoring and another using Quantum Monte Carlo Integration (QMCI) [80] to accelerate motif detection in large biological sequences. Both rely on Quantum Amplitude Amplification [81] to achieve a theoretical quadratic speedup over classical scanning, with the QMCI further reducing scoring complexity. The work shows reduced query complexity, but remains fully theoretical.

QCAM [82] uses Grover’s search and hardware-efficient quantum counting for DNA sequence similarity analysis. By decomposing sequences into overlapping k-mers and operating on k-mer representations, the framework estimates similarity scores with the expected theoretical quadratic speedup and demonstrates correct behavior on small synthetic datasets, leaving performance on real genomic workloads untested.

Chagneau et al. [83] explore quantum approaches to protein similarity by combining a quantum-generated reference sequence with quantum variants of Needleman-Wunsch, Smith-Waterman, and a conflict-graph QUBO model solved with QAOA. Using simulated backends, the study compares quantum-assisted dynamic programming with QUBO-based optimization. The conflict-graph + QAOA approach best matches BLASTP rankings, and quantum Smith-Waterman performs similarly to classical methods on short sequences. The quantum Needleman-Wunsch variant performs less reliably.

QOMIC [84] addresses the Motif identification (MI) problem by reformulating network motif counting as a binary optimization problem encoded into a Hamiltonian. To overcome qubit limitations, large biological networks are partitioned into smaller subgraphs and QAOA runs on each partition via simulation. Tested on synthetic networks and human transcriptional regulatory networks, QOMIC outperforms classical tools by identifying more valid motifs, particularly in large or dense graphs.

## Transcriptomics and Gene Expression Analysis

Dabba et al. [85] introduce a quantum-inspired extension of the Moth Flame Optimization (MFO) algorithm [86] to enhance feature selection in high-dimensional microarray data. The quantum-enhanced MFO improves exploration of the search space and mitigates premature convergence. The selected microarray gene subsets are classified with an SVM, and across all datasets, QMFO-SVM outperforms classical MFO and other baselines in accuracy and error rate.

Swathi et al. [87] classify cancer gene expression profiles by introducing a Quantum Ant Lion Optimization (QAL). It is a quantum-inspired feature-selection method that uses qubit-based probabilistic search to improve exploration of noisy, high-dimensional gene-expression data. Tested on standard cancer microarray datasets and paired with an SVM classifier, QAL achieves higher accuracy and better precision-recall performance than classical ALO and deep learning models.

QDCRNet [88] proposes a hybrid quantum-classical framework for virus detection using gene-expression data from GEO [89]. It incorporates classical preprocessing and feature selection, followed by a quantum dilated convolutional layer and GRU-based classifier. Tested on COVID-19 and leukemia datasets, the model’s accuracy surpasses classical baselines. However, all quantum elements were executed on simulators.

## Multi-Omics Data Integration

MQML [90] performs lung adenocarcinoma classification and biomarker discovery using integrated multi-omics data. After classical normalization and statistical feature reduction, compact DNA-methylation, miRNA, and mRNA feature vectors are encoded into quantum states and processed through QNN. The model shows high classification performance and identifies biologically plausible biomarkers, though results are based on simulated circuits and affected by dataset imbalance.

The Quantum Fuzzy C-Means (QFCM) [91] extends classical FCM [92] by incorporating quantum-based similarity calculations for clustering large soybean protein sequences. The sequences are transformed into fixed-length numerical feature vectors. After normalizing and encoding, QFCM computes similarity using quantum vector projection. QFCM achieves more compact and better-separated clusters than classical FCM, indicating potential benefits for biological sequence analysis.

Mohammed and Ali [93] combine classical K-means clustering with Quantum Cat Swarm Optimization (QCSO) for feature selection and SVM classification of cancer subtypes. QCSO uses a small qubit circuit to optimize feature masks without encoding omics data into quantum states. The quantum-enhanced feature selection yields substantially improved classification accuracy compared to classical baselines on a multi-omics dataset.

## Protein Design and Engineering

Khatami et al. [94] present a Grover’s search framework for protein design by formulating sequence optimization as a combinatorial search over precomputed pairwise energy tables and distance-weighted interactions. Three models are evaluated: a simplified 2D lattice system, a more realistic variant with distance-dependent energies, and a reduced HP model suitable for NISQ devices. Simulations using matrix-product-state backends [95] scale to over 200 qubits and demonstrate the expected quadratic speedup of Grover’s algorithm. A 7-qubit hardware test shows reduced accuracy due to noise but confirms feasibility for small instances.

Irbäck et al. [96] reformulate protein design under the HP 2D lattice model as a QUBO problem solvable via quantum annealing. Hybrid quantum-classical runs reliably recover optimal sequences across benchmarks, while pure annealer performance remains effective only for small sequences because of noise and embedding limitations.

## Phylogenomics and Evolutionary Analysis

NMcutDA [97] is a quantum-inspired phylogenetic reconstruction method that formulates tree building as a normalized graph-cut problem optimized on a Digital Annealer. Protein sequences are converted into a similarity matrix using BLASTP and BLOSUM62 scores, which is then treated as a weighted graph. The bipartitioning step is encoded as a QUBO, and the annealer performs iterative bipartitions to construct the final tree. NMcutDA achieves lower cut scores and higher reconstruction accuracy than classical clustering and outperforms standard phylogenetic methods, especially for highly divergent sequences, though performance declines for closely related ones.

Table 2. Summary of Qualitative Analysis across the Reviewed Studies.

| **Domain** | **Study** | **Year** | **Hybrid** | **Simulator** | **Hardware** |
| --- | --- | --- | --- | --- | --- |
| Genomics and Genome Assembly | [1] | 2021 | ✓ | ✓ | ✓ |
|  | [2] | 2021 | ✓ | ✓ | ✓ |
|  | [3] | 2022 | ✓ |  | ✓ |
|  | [4] | 2023 | ✓ | ✓ |  |
|  | [5] | 2023 | ✓ | ✓ |  |
|  | [7] | 2023 | ✓ | ✓ |  |
|  | [6] | 2024 | ✓ |  | ✓ |
| Classification and Machine Learning in Bioinformatics | [8] | 2023 | Quantum-Inspired | ✓ |  |
|  | [9] | 2024 | Quantum-Inspired | ✓ |  |
|  | [11] | 2024 | ✓ | ✓ |  |
|  | [12] | 2024 | ✓ | ✓ |  |
|  | [17] | 2024 | ✓ | ✓ |  |
|  | [21] | 2024 | ✓ | ✓ |  |
|  | [13] | 2025 | ✓ | ✓ |  |
|  | [14] | 2025 | ✓ | ✓ |  |
|  | [16] | 2025 | ✓ | ✓ |  |
|  | [19] | 2025 | ✓ | ✓ |  |
|  | [24] | 2025 | ✓ | ✓ |  |
| Drug Discovery and Molecular Interaction Modeling | [25] | 2021 | ✓ | ✓ | ✓ |
|  | [27] | 2022 | ✓ | ✓ |  |
|  | [28] | 2023 | ✓ | ✓ | ✓ |
|  | [31] | 2023 | ✓ | ✓ | ✓ |
|  | [32] | 2023 | ✓ | ✓ |  |
|  | [30] | 2024 | ✓ | ✓ |  |
|  | [35] | 2024 | ✓ | ✓ |  |
|  | [36] | 2024 | ✓ | ✓ |  |
|  | [37] | 2024 | Fully Quantum | ✓ |  |
|  | [38] | 2024 | ✓ | ✓ |  |
|  | [41] | 2024 | Fully Quantum | ✓ | ✓ |
|  | [42] | 2024 | Fully Quantum | ✓ |  |
|  | [39] | 2025 | Fully Quantum | ✓ | ✓ |
|  | [44] | 2025 | Fully Quantum | Purely Theoretical | |
|  | [45] | 2025 | ✓ | ✓ |  |
| Proteomics and Protein Structure Prediction | [46] | 2021 | Quantum-Inspired | ✓ |  |
|  | [48] | 2021 | Fully Quantum | ✓ |  |
|  | [49] | 2021 | ✓ | ✓ |  |
|  | [50] | 2021 | ✓ | ✓ | ✓ |
|  | [52] | 2022 | Quantum-Inspired | ✓ |  |
|  | [53] | 2022 | ✓ | ✓ | ✓ |
|  | [57] | 2022 | Fully Quantum | ✓ |  |
|  | [58] | 2022 | ✓ |  | ✓ |
|  | [60] | 2023 | ✓ | ✓ |  |
|  | [62] | 2023 | ✓ | ✓ |  |
|  | [69] | 2023 | ✓ | ✓ | ✓ |
|  | [63] | 2024 | ✓ | ✓ | ✓ |
|  | [68] | 2024 | Fully Quantum | ✓ |  |
|  | [65] | 2025 | ✓ | ✓ |  |
| Gene Regulation and Network Biology | [71] | 2023 | Fully Quantum | ✓ | ✓ |
|  | [74] | 2023 | ✓ | ✓ |  |
|  | [75] | 2024 | ✓ | ✓ |  |
|  | [77] | 2024 | Quantum-Inspired | ✓ |  |
|  | [76] | 2025 | ✓ | ✓ |  |
| Sequence and Motif Analysis | [79] | 2023 | Fully Quantum | Purely Theoretical | |
|  | [82] | 2023 | Fully Quantum | ✓ |  |
|  | [83] | 2024 | ✓ | ✓ |  |
|  | [84] | 2025 | ✓ | ✓ |  |
| Transcriptomics and Gene Expression Analysis | [85] | 2021 | Quantum-Inspired | ✓ |  |
|  | [87] | 2023 | Quantum-Inspired | ✓ |  |
|  | [88] | 2025 | ✓ | ✓ |  |
| Multi-Omics Data Integration | [90] | 2024 | ✓ | ✓ |  |
|  | [91] | 2024 | Quantum-Inspired | ✓ |  |
|  | [93] | 2024 | ✓ | ✓ |  |
| Protein Design and Engineering | [94] | 2023 | Fully Quantum | ✓ | ✓ |
|  | [96] | 2024 | ✓ |  | ✓ |
| Phylogenomic and Evolutionary Analysis | [97] | 2023 | Quantum-Inspired | ✓ |  |

# References

[1] A. Sarkar, Z. Al-Ars, and K. Bertels, “QuASeR: Quantum Accelerated de novo DNA sequence reconstruction,” *PLOS ONE*, vol. 16, no. 4, p. e0249850, Apr. 2021, doi: 10.1371/journal.pone.0249850.

[2] A. S. Boev *et al.*, “Genome assembly using quantum and quantum-inspired annealing,” *Sci Rep*, vol. 11, no. 1, p. 13183, Jun. 2021, doi: 10.1038/s41598-021-88321-5.

[3] K. Nałęcz-Charkiewicz and R. M. Nowak, “Algorithm for DNA sequence assembly by quantum annealing,” *BMC Bioinformatics*, vol. 23, no. 1, p. 122, Apr. 2022, doi: 10.1186/s12859-022-04661-7.

[4] J.-K. Fang *et al.*, “Divide-and-Conquer Quantum Algorithm for Hybrid $de novo$ Genome Assembly of Short and Long Reads,” *PRX Life*, vol. 2, no. 2, p. 023006, Apr. 2024, doi: 10.1103/PRXLife.2.023006.

[5] G. D. Varsamis *et al.*, “Quantum algorithm for *de novo* DNA sequence assembly based on quantum walks on graphs,” *Biosystems*, vol. 233, p. 105037, Nov. 2023, doi: 10.1016/j.biosystems.2023.105037.

[6] Y. Chen, J.-H. Huang, Y. Sun, Y. Zhang, Y. Li, and X. Xu, “Haplotype-resolved assembly of diploid and polyploid genomes using quantum computing,” *Cell Reports Methods*, vol. 4, no. 5, May 2024, doi: 10.1016/j.crmeth.2024.100754.

[7] G. D. Varsamis *et al.*, “Quantum gate algorithm for reference-guided DNA sequence alignment,” *Computational Biology and Chemistry*, vol. 107, p. 107959, Dec. 2023, doi: 10.1016/j.compbiolchem.2023.107959.

[8] R. Ahmad, M. Awais, N. Kausar, U. Tariq, J.-H. Cha, and J. Balili, “Leukocytes Classification for Leukemia Detection Using Quantum Inspired Deep Feature Selection,” *Cancers*, vol. 15, no. 9, p. 2507, Jan. 2023, doi: 10.3390/cancers15092507.

[9] A. Darolia, R. S. Chhillar, M. Alhussein, S. Dalal, K. Aurangzeb, and U. K. Lilhore, “Enhanced cardiovascular disease prediction through self-improved Aquila optimized feature selection in quantum neural network & LSTM model,” *Front. Med.*, vol. 11, Jun. 2024, doi: 10.3389/fmed.2024.1414637.

[10] L. Abualigah, D. Yousri, M. Abd Elaziz, A. A. Ewees, M. A. A. Al-qaness, and A. H. Gandomi, “Aquila Optimizer: A novel meta-heuristic optimization algorithm,” *Computers & Industrial Engineering*, vol. 157, p. 107250, Jul. 2021, doi: 10.1016/j.cie.2021.107250.

[11] S. GenÇ, “Performance Analysis of Quantum and Classical Machine Learning Models for Feature Selection and Classification of the Diabetes Health Indicators Dataset,” in *2024 8th International Artificial Intelligence and Data Processing Symposium (IDAP)*, Sep. 2024, pp. 1–7. doi: 10.1109/IDAP64064.2024.10710904.

[12] U. Ullah, D. Maheshwari, C. Castillo Olea, and B. Garcia Zapirain, “Sarcopenia risk prediction and feature selection by using quantum machine learning algorithms,” *Quantum Mach. Intell.*, vol. 6, no. 2, p. 80, Nov. 2024, doi: 10.1007/s42484-024-00218-4.

[13] L. M. Kunjachen and R. Kavitha, “Dynamic feature selection and quantum representation for precise heart disease prediction: Quantum-HeartDiseaseNet approach,” *Computer Methods in Biomechanics and Biomedical Engineering*, vol. 0, no. 0, pp. 1–22, doi: 10.1080/10255842.2025.2456990.

[14] M. N, K. R, D. V, and S. S, “Modified quantum dilated convolutional neural network for cancer prediction using gene expression data,” *Comput Methods Biomech Biomed Engin*, pp. 1–13, May 2025, doi: 10.1080/10255842.2025.2502816.

[15] Y. Chen, “Quantum Dilated Convolutional Neural Networks,” *IEEE Access*, vol. 10, pp. 20240–20246, 2022, doi: 10.1109/ACCESS.2022.3152213.

[16] E. Akpinar, B. Hangun, M. Oduncuoglu, O. Altun, O. Eyecioglu, and Z. Yalcin, “Quantum-Enhanced Classification of Brain Tumors Using DNA Microarray Gene Expression Profiles,” in *2025 IEEE Computer Society Annual Symposium on VLSI (ISVLSI)*, Jul. 2025, pp. 1–6. doi: 10.1109/ISVLSI65124.2025.11130207.

[17] P.-N. Nguyen, “Biomarker discovery with quantum neural networks: a case-study in CTLA4-activation pathways,” *BMC Bioinformatics*, vol. 25, no. 1, p. 149, Apr. 2024, doi: 10.1186/s12859-024-05755-0.

[18] J. N. Weinstein *et al.*, “The cancer genome atlas pan-cancer analysis project,” *Nature genetics*, vol. 45, no. 10, pp. 1113–1120, 2013.

[19] A. Astuti, P.-K. Shih, S.-C. Lee, V. R. Mekala, E. B. Wijaya, and K.-L. Ng, “Use of hybrid quantum-classical algorithms for enhancing biomarker classification,” *PLOS ONE*, vol. 20, no. 7, p. e0327928, Jul. 2025, doi: 10.1371/journal.pone.0327928.

[20] T. Hur, I. F. Araujo, and D. K. Park, “Neural quantum embedding: Pushing the limits of quantum supervised learning,” *Phys. Rev. A*, vol. 110, no. 2, p. 022411, Aug. 2024, doi: 10.1103/PhysRevA.110.022411.

[21] D. Kundu, A. Ghosh, S. Ekambaram, J. Wang, N. Dokholyan, and S. Ghosh, “Application of Quantum Tensor Networks for Protein Classification,” in *Proceedings of the Great Lakes Symposium on VLSI 2024*, in GLSVLSI ’24. New York, NY, USA: Association for Computing Machinery, Jun. 2024, pp. 132–137. doi: 10.1145/3649476.3658701.

[22] H.-M. Rieser, F. Köster, and A. P. Raulf, “Tensor networks for quantum machine learning,” *Proceedings of the Royal Society A: Mathematical, Physical and Engineering Sciences*, vol. 479, no. 2275, p. 20230218, Jul. 2023, doi: 10.1098/rspa.2023.0218.

[23] The UniProt Consortium, “UniProt: the Universal Protein Knowledgebase in 2023,” *Nucleic Acids Res*, vol. 51, no. D1, pp. D523–D531, Jan. 2023, doi: 10.1093/nar/gkac1052.

[24] P. Choppara and B. Lokesh, “Leveraging Quantum LSTM for High-Accuracy Prediction of Viral Mutations,” *IEEE Access*, vol. 13, pp. 25282–25300, 2025, doi: 10.1109/ACCESS.2025.3539337.

[25] J. Li, R. O. Topaloglu, and S. Ghosh, “Quantum Generative Models for Small Molecule Drug Discovery,” *IEEE Transactions on Quantum Engineering*, vol. 2, pp. 1–8, 2021, doi: 10.1109/TQE.2021.3104804.

[26] I. Goodfellow *et al.*, “Generative adversarial networks,” *Commun. ACM*, vol. 63, no. 11, pp. 139–144, Oct. 2020, doi: 10.1145/3422622.

[27] J. Li and S. Ghosh, “Scalable Variational Quantum Circuits for Autoencoder-based Drug Discovery,” in *2022 Design, Automation & Test in Europe Conference & Exhibition (DATE)*, Mar. 2022, pp. 340–345. doi: 10.23919/DATE54114.2022.9774564.

[28] S. Mensa, E. Sahin, F. Tacchino, P. Kl Barkoutsos, and I. Tavernelli, “Quantum machine learning framework for virtual screening in drug discovery: a prospective quantum advantage,” *Mach. Learn.: Sci. Technol.*, vol. 4, no. 1, p. 015023, Feb. 2023, doi: 10.1088/2632-2153/acb900.

[29] P. Rebentrost, M. Mohseni, and S. Lloyd, “Quantum Support Vector Machine for Big Data Classification,” *Phys. Rev. Lett.*, vol. 113, no. 13, p. 130503, Sep. 2014, doi: 10.1103/PhysRevLett.113.130503.

[30] M. A. Dorsey *et al.*, “Near-Term Quantum Classification Algorithms Applied to Antimalarial Drug Discovery,” *J. Chem. Inf. Model.*, vol. 64, no. 15, pp. 5922–5930, Aug. 2024, doi: 10.1021/acs.jcim.4c00953.

[31] B. Lau *et al.*, “Insights from incorporating quantum computing into drug design workflows,” *Bioinformatics*, vol. 39, no. 1, p. btac789, Jan. 2023, doi: 10.1093/bioinformatics/btac789.

[32] L. Dong *et al.*, “Prediction of Protein-Ligand Binding Affinity by a Hybrid Quantum-Classical Deep Learning Algorithm,” *Advanced Quantum Technologies*, vol. 6, no. 9, p. 2300107, 2023, doi: 10.1002/qute.202300107.

[33] T. N. Kipf and M. Welling, “Semi-Supervised Classification with Graph Convolutional Networks,” Feb. 22, 2017, *arXiv*: arXiv:1609.02907. doi: 10.48550/arXiv.1609.02907.

[34] K. Xu, W. Hu, J. Leskovec, and S. Jegelka, “How Powerful are Graph Neural Networks?,” Feb. 22, 2019, *arXiv*: arXiv:1810.00826. doi: 10.48550/arXiv.1810.00826.

[35] L. Domingo *et al.*, “A Hybrid Quantum-classical Fusion Neural Network to Improve Protein-ligand Binding Affinity Predictions for Drug Discovery,” in *2024 IEEE International Conference on Quantum Computing and Engineering (QCE)*, Sep. 2024, pp. 126–131. doi: 10.1109/QCE60285.2024.10265.

[36] M. Avramouli, I. K. Savvas, A. Vasilaki, A. Tsipourlianos, and G. Garani, “Hybrid Quantum Neural Network Approaches to Protein–Ligand Binding Affinity Prediction,” *Mathematics*, vol. 12, no. 15, p. 2372, Jan. 2024, doi: 10.3390/math12152372.

[37] P. CHOPPARA and B. LOKESH, “Quantum Machine Learning for Prediction of Compound-Protein Interactions in Drug Discovery,” in *2024 12th International Conference on Intelligent Systems and Embedded Design (ISED)*, Dec. 2024, pp. 1–6. doi: 10.1109/ISED63599.2024.10956316.

[38] S. Ganguly, V. Chandilkar, P. Jain, and L. G. A. Bertel, “Quantum Graph Neural Networks Based Protein-Ligand Classification,” in *Artificial Intelligence and Knowledge Processing*, H. K, R. V. Rodriguez, M. Rege, V. Piuri, G. Xu, and K.-L. Ong, Eds., Cham: Springer Nature Switzerland, 2024, pp. 146–159.

[39] I. Liliopoulos *et al.*, “Quantum algorithm for protein-ligand docking sites identification in the interaction space,” *J Comput Aided Mol Des*, vol. 39, no. 1, p. 40, Jul. 2025, doi: 10.1007/s10822-025-00620-5.

[40] S. Pattanayak, *Quantum Machine Learning with Python: Using Cirq from Google Research and IBM Qiskit*. Berkeley, CA: Apress, 2021. doi: 10.1007/978-1-4842-6522-2.

[41] G. Lancellotti, G. Accordi, and G. Palermo, “An Experimental Approach to Quantum Molecular Docking,” in *2024 IEEE International Conference on Quantum Computing and Engineering (QCE)*, Sep. 2024, pp. 512–518. doi: 10.1109/QCE60285.2024.00066.

[42] Q.-M. Ding, Y.-M. Huang, and X. Yuan, “Molecular docking via quantum approximate optimization algorithm,” *Phys. Rev. Appl.*, vol. 21, no. 3, p. 034036, Mar. 2024, doi: 10.1103/PhysRevApplied.21.034036.

[43] P. Chandarana *et al.*, “Digitized-counterdiabatic quantum approximate optimization algorithm,” *Phys. Rev. Res.*, vol. 4, no. 1, p. 013141, Feb. 2022, doi: 10.1103/PhysRevResearch.4.013141.

[44] S. Surya Prakash, P. Banu Priya, S. Someneni, Udendhran, and A. Banerjee, “Drug Discovery Using Variational Quantum EigenSolver,” in *Proceedings of Fourth International Conference on Computing and Communication Networks*, A. Kumar, A. Swaroop, and P. Shukla, Eds., Singapore: Springer Nature, 2025, pp. 691–703. doi: 10.1007/978-981-96-3942-7_51.

[45] T. Bikku, K. K. Malligunta, S. Thota, and P. P. Surapaneni, “Improved Quantum Algorithm: A Crucial Stepping Stone in Quantum-Powered Drug Discovery,” *J. Electron. Mater.*, vol. 54, no. 5, pp. 3434–3443, May 2025, doi: 10.1007/s11664-024-11275-7.

[46] W.-W. Mao, L.-H. Lu, and Y.-Q. Li, “Quantum model for understanding protein misfolding behavior—phase diagram and manual intervention,” *Sci. China Phys. Mech. Astron.*, vol. 64, no. 6, p. 260011, Apr. 2021, doi: 10.1007/s11433-020-1691-3.

[47] G. Lindblad, “On the generators of quantum dynamical semigroups,” *Commun.Math. Phys.*, vol. 48, no. 2, pp. 119–130, Jun. 1976, doi: 10.1007/BF01608499.

[48] R. Wong and W.-L. Chang, “Quantum Speedup for Protein Structure Prediction,” *IEEE Transactions on NanoBioscience*, vol. 20, no. 3, pp. 323–330, Jul. 2021, doi: 10.1109/TNB.2021.3065051.

[49] Z. Hong, J. Wang, X. Qu, X. Zhu, J. Liu, and J. Xiao, “Quantum Convolutional Neural Network on Protein Distance Prediction,” in *2021 International Joint Conference on Neural Networks (IJCNN)*, Jul. 2021, pp. 1–8. doi: 10.1109/IJCNN52387.2021.9533405.

[50] A. Robert, P. K. Barkoutsos, S. Woerner, and I. Tavernelli, “Resource-efficient quantum algorithm for protein folding,” *npj Quantum Inf*, vol. 7, no. 1, p. 38, Feb. 2021, doi: 10.1038/s41534-021-00368-4.

[51] P. K. Barkoutsos, G. Nannicini, A. Robert, I. Tavernelli, and S. Woerner, “Improving Variational Quantum Optimization using CVaR,” *Quantum*, vol. 4, p. 256, Apr. 2020, doi: 10.22331/q-2020-04-20-256.

[52] M. Atari and N. Majd, “2D HP protein folding using quantum genetic algorithm,” in *2022 27th International Computer Conference, Computer Society of Iran (CSICC)*, Feb. 2022, pp. 1–8. doi: 10.1109/CSICC55295.2022.9780478.

[53] P. A. M. Casares, R. Campos, and M. A. Martin-Delgado, “QFold: quantum walks and deep learning to solve protein folding,” *Quantum Sci. Technol.*, vol. 7, no. 2, p. 025013, Mar. 2022, doi: 10.1088/2058-9565/ac4f2f.

[54] E. Alcaide, “MiniFold: a DeepLearning-based Mini Protein Folding Engine,” *GitHub repository*. GitHub, 2019. doi: 10.5281/zenodo.3774491.

[55] J. Lemieux, B. Heim, D. Poulin, K. Svore, and M. Troyer, “Efficient Quantum Walk Circuits for Metropolis-Hastings Algorithm,” *Quantum*, vol. 4, p. 287, Jun. 2020, doi: 10.22331/q-2020-06-29-287.

[56] N. Metropolis, A. W. Rosenbluth, M. N. Rosenbluth, A. H. Teller, and E. Teller, “Equation of State Calculations by Fast Computing Machines,” *J. Chem. Phys.*, vol. 21, no. 6, pp. 1087–1092, Jun. 1953, doi: 10.1063/1.1699114.

[57] R. Wong and W.-L. Chang, “Fast quantum algorithm for protein structure prediction in hydrophobic-hydrophilic model,” *Journal of Parallel and Distributed Computing*, vol. 164, pp. 178–190, Jun. 2022, doi: 10.1016/j.jpdc.2022.03.011.

[58] A. Irbäck, L. Knuthson, S. Mohanty, and C. Peterson, “Folding lattice proteins with quantum annealing,” *Phys. Rev. Res.*, vol. 4, no. 4, p. 043013, Oct. 2022, doi: 10.1103/PhysRevResearch.4.043013.

[59] S. Kirkpatrick, C. D. Gelatt, and M. P. Vecchi, “Optimization by Simulated Annealing,” *Science*, vol. 220, no. 4598, pp. 671–680, May 1983, doi: 10.1126/science.220.4598.671.

[60] A. I. Muscalagiu, “Quantum Reinforcement Learning in Protein Folding,” in *2023 25th International Symposium on Symbolic and Numeric Algorithms for Scientific Computing (SYNASC)*, Sep. 2023, pp. 316–323. doi: 10.1109/SYNASC61333.2023.00053.

[61] D. Dong, C. Chen, H. Li, and T.-J. Tarn, “Quantum Reinforcement Learning,” *IEEE Transactions on Systems, Man, and Cybernetics, Part B (Cybernetics)*, vol. 38, no. 5, pp. 1207–1220, Oct. 2008, doi: 10.1109/TSMCB.2008.925743.

[62] G. D. Varsamis and I. G. Karafyllidis, “A quantum walks assisted algorithm for peptide and protein folding prediction,” *Biosystems*, vol. 223, p. 104822, Jan. 2023, doi: 10.1016/j.biosystems.2022.104822.

[63] J. V. Pamidimukkala, S. Bopardikar, A. Dakshinamoorthy, A. Kannan, K. Dasgupta, and S. Senapati, “Protein Structure Prediction with High Degrees of Freedom in a Gate-Based Quantum Computer,” *J. Chem. Theory Comput.*, vol. 20, no. 22, pp. 10223–10234, Nov. 2024, doi: 10.1021/acs.jctc.4c00848.

[64] I. I. Cplex and others, “V12. 1: User’s Manual for CPLEX,” *International business machines corporation*, vol. 46, no. 53, p. 157, 2009.

[65] Y. Wang and X. Zhou, “Efficient quantum algorithm for lattice protein folding,” *Quantum Sci. Technol.*, vol. 10, no. 1, p. 015056, Dec. 2024, doi: 10.1088/2058-9565/ada08e.

[66] J. R. McClean, S. Boixo, V. N. Smelyanskiy, R. Babbush, and H. Neven, “Barren plateaus in quantum neural network training landscapes,” *Nat Commun*, vol. 9, no. 1, p. 4812, Nov. 2018, doi: 10.1038/s41467-018-07090-4.

[67] S. Wang *et al.*, “Noise-induced barren plateaus in variational quantum algorithms,” *Nat Commun*, vol. 12, no. 1, p. 6961, Nov. 2021, doi: 10.1038/s41467-021-27045-6.

[68] S. Bhuvaneswari, R. Deepakraj, S. Urooj, N. Sharma, and N. Pathak, “Computational Analysis: Unveiling the Quantum Algorithms for Protein Analysis and Predictions,” *IEEE Access*, vol. 11, pp. 94023–94033, 2023, doi: 10.1109/ACCESS.2023.3310812.

[69] P. Chandarana, N. N. Hegade, I. Montalban, E. Solano, and X. Chen, “Digitized Counterdiabatic Quantum Algorithm for Protein Folding,” *Phys. Rev. Appl.*, vol. 20, no. 1, p. 014024, Jul. 2023, doi: 10.1103/PhysRevApplied.20.014024.

[70] S. Miyazawa and R. L. Jernigan, “Residue – Residue Potentials with a Favorable Contact Pair Term and an Unfavorable High Packing Density Term, for Simulation and Threading,” *Journal of Molecular Biology*, vol. 256, no. 3, pp. 623–644, Mar. 1996, doi: 10.1006/jmbi.1996.0114.

[71] F. M. Weidner *et al.*, “Leveraging quantum computing for dynamic analyses of logical networks in systems biology,” *Patterns*, vol. 4, no. 3, Mar. 2023, doi: 10.1016/j.patter.2023.100705.

[72] G. Brassard, P. HØyer, and A. Tapp, “Quantum counting,” in *Automata, Languages and Programming*, K. G. Larsen, S. Skyum, and G. Winskel, Eds., Berlin, Heidelberg: Springer, 1998, pp. 820–831. doi: 10.1007/BFb0055105.

[73] A. E. Brisebois, J. Broderick, Z. Khatooni, H. L. Wilson, S. Rayan, and G. Broderick, “Identifying Protein Co-regulatory Network Logic by Solving B-SAT Problems through Gate-based Quantum Computing,” Jul. 17, 2025, *arXiv*: arXiv:2504.09365. doi: 10.48550/arXiv.2504.09365.

[74] C. Roman-Vicharra and J. J. Cai, “Quantum gene regulatory networks,” *npj Quantum Inf*, vol. 9, no. 1, p. 67, Jul. 2023, doi: 10.1038/s41534-023-00740-6.

[75] H. Wang, “Q-TetoFormer: A New Gene Regulatory Network Prediction Method Based on Quantum Computing and Transformer,” in *2024 IEEE International Conference on Bioinformatics and Biomedicine (BIBM)*, Dec. 2024, pp. 471–474. doi: 10.1109/BIBM62325.2024.10822209.

[76] D. Konar, N. Sreekumar, R. Jiang, and V. Aggarwal, “Alz-QNet: A quantum regression network for studying Alzheimer’s gene interactions,” *Computers in Biology and Medicine*, vol. 196, p. 110837, Sep. 2025, doi: 10.1016/j.compbiomed.2025.110837.

[77] H. Saarinen, M. Goldsmith, R.-S. Wang, J. Loscalzo, and S. Maniscalco, “Disease gene prioritization with quantum walks,” *Bioinformatics*, vol. 40, no. 8, p. btae513, Aug. 2024, doi: 10.1093/bioinformatics/btae513.

[78] E. Farhi and S. Gutmann, “Quantum computation and decision trees,” *Phys. Rev. A*, vol. 58, no. 2, pp. 915–928, Aug. 1998, doi: 10.1103/PhysRevA.58.915.

[79] K. Miyamoto, N. Yamamoto, and Y. Sakakibara, “Quantum Algorithm for Position Weight Matrix Matching,” *IEEE Transactions on Quantum Engineering*, vol. 4, pp. 1–14, 2023, doi: 10.1109/TQE.2023.3293562.

[80] A. Montanaro, “Quantum speedup of Monte Carlo methods,” *Proceedings of the Royal Society A: Mathematical, Physical and Engineering Sciences*, vol. 471, no. 2181, p. 20150301, Sep. 2015, doi: 10.1098/rspa.2015.0301.

[81] G. Brassard, P. Hoyer, M. Mosca, and A. Tapp, “Quantum Amplitude Amplification and Estimation,” vol. 305, 2002, pp. 53–74. doi: 10.1090/conm/305/05215.

[82] J. Balewski, D. Camps, K. Klymko, and A. Tritt, “Efficient Quantum Counting and Quantum Content-Addressable Memory for DNA Similarity,” in *2023 IEEE International Conference on Quantum Computing and Engineering (QCE)*, Sep. 2023, pp. 378–384. doi: 10.1109/QCE57702.2023.00050.

[83] “Quantum algorithm for bioinformatics to compute the similarity between proteins - Chagneau - 2024 - IET Quantum Communication - Wiley Online Library.” Accessed: Nov. 13, 2025. [Online]. Available: https://ietresearch.onlinelibrary.wiley.com/doi/full/10.1049/qtc2.12098

[84] H. M. Ngo, T. Khatib, M. T. Thai, and T. Kahveci, “QOMIC: quantum optimization for motif identification,” *Bioinformatics Advances*, vol. 5, no. 1, p. vbae208, Jan. 2025, doi: 10.1093/bioadv/vbae208.

[85] A. Dabba, A. Tari, and S. Meftali, “Hybridization of Moth flame optimization algorithm and quantum computing for gene selection in microarray data,” *J Ambient Intell Human Comput*, vol. 12, no. 2, pp. 2731–2750, Feb. 2021, doi: 10.1007/s12652-020-02434-9.

[86] S. Mirjalili, “Moth-flame optimization algorithm: A novel nature-inspired heuristic paradigm,” *Knowledge-Based Systems*, vol. 89, pp. 228–249, Nov. 2015, doi: 10.1016/j.knosys.2015.07.006.

[87] K. SWATHI and S. KODUKULA, “A NOVEL APPROACH BASED ON FEATURE SELECTION AND GENE CLASSIFICATION USING SUPPORT VECTOR MACHINES AND QUANTUM ANT LION OPTIMIZATION,” *Journal of Theoretical and Applied Information Technology*, vol. 101, no. 22, 2023.

[88] S. Karthi, T. Ramalingam, R. Iyswarya, and D. A. Kumar, “QDCRNet: Quantum dilated convolutional recurrent network for virus detection using gene expression data,” *Computational Biology and Chemistry*, vol. 120, p. 108625, Feb. 2026, doi: 10.1016/j.compbiolchem.2025.108625.

[89] “GEO Accession viewer.” Accessed: Nov. 14, 2025. [Online]. Available: https://www.ncbi.nlm.nih.gov/geo/query/acc.cgi?acc=GSE164805

[90] M. K. Saggi and S. Kais, “MQML: Multi-Omic Quantum Machine Learning Based Cancer Classification, Biomarker Identification in Human Lung Adenocarcinoma,” in *2024 IEEE International Conference on Quantum Computing and Engineering (QCE)*, Sep. 2024, pp. 1713–1720. doi: 10.1109/QCE60285.2024.00200.

[91] S. S. V. D. Rangoju, K. Garg, R. Dandi, O. P. Patel, and N. Bharill, “Soybean Genome Clustering Using Quantum-Based Fuzzy C-Means Algorithm,” in *Neural Information Processing*, B. Luo, L. Cheng, Z.-G. Wu, H. Li, and C. Li, Eds., Singapore: Springer Nature, 2024, pp. 83–94. doi: 10.1007/978-981-99-8070-3_7.

[92] J. C. BEZDEK, “Fuzzy-Mathematics in Pattern Classification.,” Ph.D., Cornell University, United States -- New York, 1973. Accessed: Nov. 15, 2025. [Online]. Available: https://www.proquest.com/docview/302663706/citation/4ADBC54C54744FA7PQ/1

[93] M. Mohammed and A. Ali, “Enhanced Cancer Subclassification Using Multi-Omics Clustering and Quantum Cat Swarm Optimization,” *Iraqi Journal for Computer Science and Mathematics*, vol. 5, no. 3, Jan. 2024, doi: 10.52866/ijcsm.2024.05.03.035.

[94] M. H. Khatami, U. C. Mendes, N. Wiebe, and P. M. Kim, “Gate-based quantum computing for protein design,” *PLOS Computational Biology*, vol. 19, no. 4, p. e1011033, Apr. 2023, doi: 10.1371/journal.pcbi.1011033.

[95] G. Vidal, “Efficient Classical Simulation of Slightly Entangled Quantum Computations,” *Phys. Rev. Lett.*, vol. 91, no. 14, p. 147902, Oct. 2003, doi: 10.1103/PhysRevLett.91.147902.

[96] A. Irbäck, L. Knuthson, S. Mohanty, and C. Peterson, “Using quantum annealing to design lattice proteins,” *Phys. Rev. Res.*, vol. 6, no. 1, p. 013162, Feb. 2024, doi: 10.1103/PhysRevResearch.6.013162.

[97] W. Onodera, N. Hara, S. Aoki, T. Asahi, and N. Sawamura, “Phylogenetic tree reconstruction via graph cut presented using a quantum-inspired computer,” *Molecular Phylogenetics and Evolution*, vol. 178, p. 107636, Jan. 2023, doi: 10.1016/j.ympev.2022.107636.
